# Interictal waking and sleep electrophysiological properties of the thalamus in focal epilepsies

**DOI:** 10.1093/braincomms/fcaf102

**Published:** 2025-03-05

**Authors:** Tommaso Biagioni, Maria Fratello, Elodie Garnier, Stanislas Lagarde, Romain Carron, Samuel Medina Villalon, Isabelle Lambert, Angela Marchi, Julia Makhalova, Agnes Trebuchon, Francesca Bonini, Didier Scavarda, Christian Benar, Fabrice Bartolomei, Francesca Pizzo

**Affiliations:** Department of Clinical and Experimental Medicine, University of Pisa, 56123 Pisa, Italy; Faculty of Medicine, The University of Queensland, Brisbane, 4072 Queensland, Australia; INSERM, INS, Inst Neurosci Syst, Aix Marseille Univ, 13005 Marseille, France; INSERM, INS, Inst Neurosci Syst, Aix Marseille Univ, 13005 Marseille, France; INSERM, INS, Inst Neurosci Syst, Aix Marseille Univ, 13005 Marseille, France; APHM, Timone Hospital, Epileptology and Cerebral Rhythmology, 13005 Marseille, France; INSERM, INS, Inst Neurosci Syst, Aix Marseille Univ, 13005 Marseille, France; APHM, Timone Hospital, Functional, and Stereotactic Neurosurgery, 13005 Marseille, France; INSERM, INS, Inst Neurosci Syst, Aix Marseille Univ, 13005 Marseille, France; APHM, Timone Hospital, Epileptology and Cerebral Rhythmology, 13005 Marseille, France; INSERM, INS, Inst Neurosci Syst, Aix Marseille Univ, 13005 Marseille, France; APHM, Timone Hospital, Sleep Unit, Epileptology and Cerebral Rhythmology, 13005 Marseille, France; APHM, Timone Hospital, Sleep Unit, Epileptology and Cerebral Rhythmology, 13005 Marseille, France; INSERM, INS, Inst Neurosci Syst, Aix Marseille Univ, 13005 Marseille, France; APHM, Timone Hospital, Epileptology and Cerebral Rhythmology, 13005 Marseille, France; Aix Marseille Univ, CNRS, CRMBM, 13005 Marseille, France; INSERM, INS, Inst Neurosci Syst, Aix Marseille Univ, 13005 Marseille, France; APHM, Timone Hospital, Epileptology and Cerebral Rhythmology, 13005 Marseille, France; INSERM, INS, Inst Neurosci Syst, Aix Marseille Univ, 13005 Marseille, France; APHM, Timone Hospital, Epileptology and Cerebral Rhythmology, 13005 Marseille, France; INSERM, INS, Inst Neurosci Syst, Aix Marseille Univ, 13005 Marseille, France; Department of Pediatric Neurosurgery, APHM, 13005 Marseille, France; INSERM, INS, Inst Neurosci Syst, Aix Marseille Univ, 13005 Marseille, France; INSERM, INS, Inst Neurosci Syst, Aix Marseille Univ, 13005 Marseille, France; APHM, Timone Hospital, Epileptology and Cerebral Rhythmology, 13005 Marseille, France; INSERM, INS, Inst Neurosci Syst, Aix Marseille Univ, 13005 Marseille, France; APHM, Timone Hospital, Epileptology and Cerebral Rhythmology, 13005 Marseille, France

**Keywords:** intracranial EEG, thalamic hyperexcitability, thalamo-cortical functional connectivity, surgical outcome, epileptogenic networks

## Abstract

Epilepsy is a cortico-subcortical network disease. Thalamo-cortical relationships in focal epilepsies, studied by stereoelectroencephalography in complex patients during pre-surgical evaluation, might help refine epilepsy surgery prognostic indicators and patient-specific treatments (i.e. thalamic deep brain stimulation). To this aim, we studied interictal thalamic traces, during rest and sleep recordings, in a cohort of 121 patients, delving into thalamo-cortical connectivity, hyperexcitability biomarkers and their correlation with treatment outcome.

We retrospectively gathered stereoelectroencephalography recordings and clinical variables from patients who underwent stereoelectroencephalography with mainly a posterior-thalamic implantation, aiming at the pulvinar. Interictal recordings during rest and sleep were analysed to detect spikes and fast ripples automatically. Functional connectivity between the thalamus and other brain regions (involved or non-involved in the epileptogenic network) was examined using linear regression analysis.

Higher thalamic hyperexcitability biomarker rates during sleep were linked to unfavourable surgical outcomes (Engel Class III/IV) compared to favourable outcomes (Engel Class I/II) (spikes: *N* = 117, *P* = 0.009, effect size = 0.25; fast ripples: *N* = 17, *P* = 0.036, effect size = 0.52). Thalamo-cortical functional connectivity analysis revealed heightened thalamic strength, particularly in the beta (*P* < 0.001, effect size = 0.38) and gamma (*P* = 0.012, effect size = 0.24) bands during sleep, among patients with poor surgical outcomes, especially with non-involved networks. Conversely, during rest, lower hyperexcitability biomarkers (spikes *r* = −0.2, *P* = 0.048; fast ripples *r* = −0.52, *P* = 0.045) and lower values of thalamic strength (delta band *r* = −0.28, *P* = 0.025; broadband *r* = −0.23, *P* = 0.01) were observed in patients with longer epilepsy duration. Furthermore, thalamic strength values during rest were lower in patients of older age (broadband *r* = −0.19, *P* = 0.045).

These findings confirmed the important role of the thalamus in focal epilepsy. According to this exploratory group-level study, thalamic recordings could potentially improve pre-surgical assessment and help identify patients who may have a less severe outcome. Additionally, diminished thalamic activity and connectivity associated with epilepsy duration and age prompt speculation on the role of thalamo-cortical interactions in ageing-related physiological and pathological processes.

## Teaser

Epilepsy is a cortico-subcortical network disorder. By analysing thalamo-cortical relationships in 121 patients through stereotactic-EEG, Biagioni *et al*. identified that higher thalamic hyperexcitability and strength functional connectivity during sleep correlates with worse surgical outcomes, while changes in thalamic strength functional connectivity during rest recordings offer insights into epilepsy duration and age effects.

## Graphical Abstract

**Graphical Abstract:**
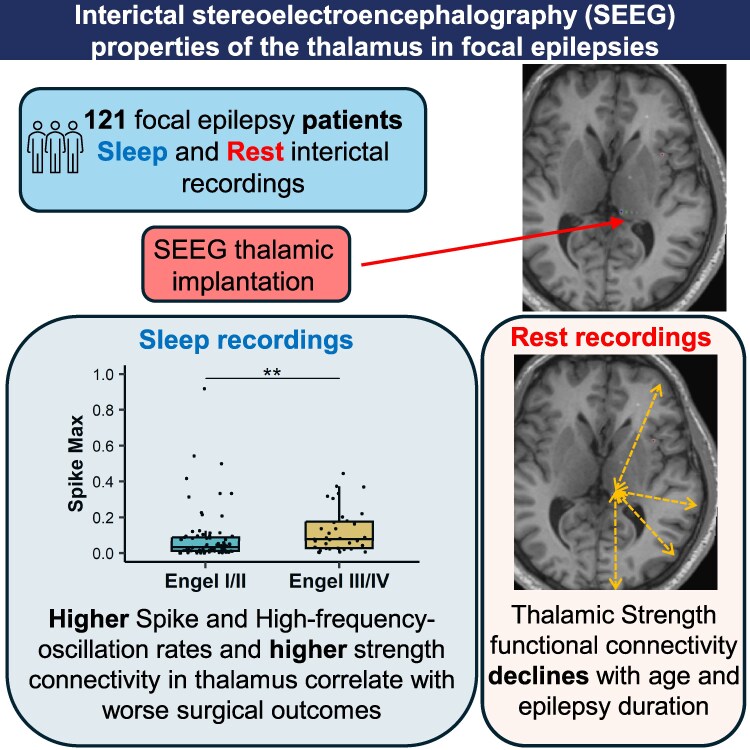

## Introduction

Epilepsy, a complex cortico-subcortical neurological disorder,^1^ presents a significant clinical challenge when it proves refractory to anti-seizure medications.

Resective surgery in some focal epilepsies could be proposed.^2^ To identify the epileptogenic network for surgical purposes, non-invasive explorations (videoEEG monitoring, MRI, positron emission tomography, magnetoencephalography and others) might not be sufficient, requiring invasive monitoring.^3^ Stereoelectroencephalography (SEEG), a minimally invasive technique consisting in implanting intracerebral electrodes, can better identify the epileptogenic zone (EZ) network by sampling simultaneously from deep and lateral brain regions.^4^

According to recent literature data, after SEEG exploration almost half of the patients are recused for surgery and in some operated patients surgery is not resolutive.^5-7^ Some other treatments are needed. In this context, thalamic deep brain stimulation (DBS) can be proposed,^8^ however, the clear mechanisms of action of this technique are still not completely understood.

SEEG offers the possibility to study the intricate relationship between the thalamus and the cortex by recording both thalamic and cortical activities during the pre-surgical evaluation of patients with drug-resistant focal epilepsy.^9-11^ Most of the studies on thalamic SEEG so far have focused on the ictal period, meaning during epileptic seizures.^9,12-14^

Thalamic involvement was observed in focal seizures in more than 80% of studied cases. The degree of thalamic involvement significantly affected the post-surgical outcome (higher involvement, higher probability of surgical failure).^13^ Functional connectivity (FC) studies showed a significant involvement of the thalamus in loss of consciousness during seizures^15^ and in seizure termination.^14-16^ These data suggest the thalamus as an interesting hub in focal epilepsy, underscoring its relevance for comprehending not only the mechanisms of epileptogenicity but also for exploring therapeutic options such as DBS in patients in whom surgery is contraindicated or has failed.

DBS^17^ is indeed a potential treatment, associated or not with Responsive Neurostimulation,^18^ for patients with drug-resistant epilepsy when resective epilepsy surgery is precluded.^19^ Interestingly, DBS acts mostly in the interictal period, but only a few studies have investigated interictal SEEG thalamic activity and a limited number of patients.^20,21^ Some of these studies tested the response on cortical regions following thalamic stimulation, showing a modification in spike rate and FC.^22-24^ Regarding cortico-cortical FC in the interictal period, it was evidenced that more extensive FC alterations, implicating the non-involved network and suggesting a more diffuse disease, were associated with poorer post-surgical prognosis.^6^ However, thalamo-cortical interictal FC is still unexplored.

The aim of this research is to understand the role of thalamus during the interictal period in focal drug-resistant epilepsy, giving the growing interest in thalamic DBS for epilepsy as a treatment for patients not candidates for surgery or after surgery failure. Considering the valuable insights into epileptogenicity, surgical outcomes and the efficacy of regulating interictal activities derived from the study of thalamic neurophysiology, we analysed the relationship between the thalamus and cortex in focal drug-resistant epilepsy, in a cohort of 121 patients, with the aim to reveal correlations with clinical data and potentially identifying prognostic factors at a group level. We examined thalamic interictal activity and thalamo-cortical interictal FC during two distinct vigilance states—waking resting state and non-rapid eye movement (NREM) sleep, which is known to significantly increase interictal epileptic activities.^25^ The deliberate choice of these states provides a nuanced understanding of the dynamic thalamo-cortical interplay across different physiological contexts.

## Materials and methods

### Patient selection and clinical data collection

#### The study design and realization adhere to the STROBE guidelines

Patients that underwent an SEEG study from January 2001 to December 2022 in the Epileptology and Cerebral Rhythmology Unit at the University Hospitals of Marseille, France were selected and included if they had at least one electrode recording from the thalamus. The implantation of the thalamus, most of the time aiming for the pulvinar medialis, is considered when a posterior temporal electrode exploring the *planum temporale* or the Heschl gyrus is needed for the definition of the EZ and/or for the identification of eloquent cortex. The safety of this procedure has been already discussed in previous studies.^13,26^ Most of the patients in this study were implanted in the pulvinar (88/121) because of the known connections of the medial pulvinar with the limbic structures, the insula and the lateral temporal cortex^11^ and because this structure is emerging as a possible site for DBS implantation.^27-30^ Moreover, the pulvinar is easily accessible when it is necessary to explore Heschl's cortex in the process of defining language lateralization during the pre-surgical evaluation.

Patient clinical data (date of birth, sex, epilepsy aetiology, EZ localization, MRI findings, age at epilepsy onset and epilepsy duration) and SEEG data (patient's age at SEEG, localization of the contact exploring the thalamus and eventual bithalamic implantation) were collected. The epilepsy type was defined as: temporal, frontal, occipital, insulo-opercular, parietal and multi-lobar (epilepsies involving more than one cerebral lobe). Finally, data concerning the treatments received during and after the SEEG, such as thermocoagulations, surgery and/or neurostimulation (vagus nerve stimulation, DBS or transcranial direct current stimulation) were also collected. The surgical outcome has been determined using the Engel classification,^31^ at the last available follow-up, both for patients who underwent surgery and those who received thermocoagulations only.

### Stereotactic-EEG recordings and interictal recordings

Interictal recordings that were temporally distant from the preceding and the following seizure by at least 2 h were selected.^6^ In addition, recordings were chosen at least 2 days after the electrode implantation surgical procedure to limit possible effects of general anaesthesia. For each patient were selected:

1. Ten minutes of ‘rest recording’: time period in which patients stay at rest with eyes closed but awake (recording performed preferably between 07:00 a.m. and 12:30 p.m.);
2. Ten minutes of ‘sleep recording’: N2-N3 sleep stages with a minimal number of arousals, possibly during the first or the second sleep cycle. The SEEG NREM recordings were selected for the presence of slow waves and spindles in frontal neocortical regions and, notably, in the thalamus.

The SEEG exploration was performed in the context of the pre-surgical evaluation of epilepsy using intracerebral multiple-contact electrodes (Dixi Medical or Alcis), consisting of 10–18 contacts with length 2 mm, diameter 0.8 mm, spaced by 1.5 mm.^13^ Signals were recorded on a 128- or 256-channel Natus system depending on the year of recording. They were sampled at 256, 512, 1024 or 2048 Hz depending on the year of recording, and recorded on a hard disk (16 bits/sample) using no digital filter. Two hardware filters were present in the acquisition procedure: a high-pass filter (cut-off frequency = 0.16 Hz at −3 dB), and an anti-aliasing low-pass filter (cut-off frequency = 97 Hz for 256 Hz sampling rate, 170 Hz for 512 Hz sampling rate, 340 Hz for 1024 Hz sampling rate or 680 Hz for 2048 Hz sampling rate). Artefacts and bad channels were excluded from the analysis.

All patients in the study have at least one SEEG electrode recording from thalamus. Postoperative computed tomography and preoperative 3D T1-weighted sequences were co-registered using GARDEL software (https://gitlab-dynamap.timone.univ-amu.fr/public_shared_tools/gardel/-/wikis/home). This software allows the precise localization of each electrode's contact on the different brain regions. The position inside the thalamus, more precisely the pulvinar when it was the case, was checked by two independent reviewers (TB, FP) in all patients. To reduce the influence of common noise across electrodes, we choose to analyse data on a bipolar montage emphasizing the differences between adjacent electrode pairs, which can enhance the detection of localized brain activity. We chose electrodes only in the grey matter or inside subcortical nuclei, avoiding contacts in the white matter, preferring to use deeper contacts for the analyses. Thus, for the thalamus, both bipolar contacts were selected within the thalamus (in the case of pulvinar implantation, both contacts were within this thalamic subnucleus).

### Brain Imaging Data Structure Anywave

The anonymized clinical data of the patients, their imaging and SEEG records were organized according to the Brain Imaging Data Structure standard, using Brain Imaging Data Structure Manager,^32^ a software that allows to explore and dynamically organize the data according to the Brain Imaging Data Structure standard whilst tracking the subject data readiness and integrity.

All signal analysis was computed using the Anywave software^33^ (open-source at http://meg.univ-amu.fr/wiki/AnyWave).

### Semi-automatic interictal activities detection

To detect interictal paroxysmal activities during rest and sleep, the Delphos detector was used, looking for spikes (all recordings) and fast ripples high frequency oscillations (HFO) (250–500 Hz) (for recordings sampled at 2048 Hz) on all channels without artefacts on a grey matter bipolar montage, excluding white matter channels (each bipolar contact analysed was selected between two contacts in the grey matter). The Delphos detector^34^ is based on the ZH0 method, which aims at flattening the frequency spectrum to enhance the fast oscillations while preserving an optimal signal-to-noise ratio at each frequency. In this study, the ripple frequencies (150–250 Hz) were not studied in order to avoid the possible interferences of physiological activities in that frequency band.^35^ A visual validation of the automatic detector was performed on sleep and rest recordings from 10 patients, focusing on the thalamus, to confirm the accuracy of spike and fast ripple detection and to ensure the applicability of the detection settings in this subcortical structure.

Because some patients had multiple bipolar channels recording the thalamus, we computed at the patient level the maximum thalamic spike rate and HFO rate, during rest and sleep (called ‘Spike Max’ and ‘HFO Max’). Furthermore, as these rates were highly variable from individual to individual, all values were normalized by the highest spike or HFO rate (across at SEEG channels) recorded in the individual patient and therefore the values are between 0 and 1.

To check the intra-patient reliability of our measures, for 10 patients, we repeated the calculation on a different sleep recording to measure the maximum thalamic spike rate, and we checked for significant differences between the two samples. For each patient, the second sleep recording (N2-N3 sleep stages) has been selected ∼1 h later than the one used for the main analyses.

### FC analysis: liner regression method (*R*^2^)

To investigate the FC between the thalamus and the other brain regions during the interictal period (rest and sleep), we computed on bipolar montage the linear correlation coefficient *R*^2^. For two time series *x*(*t*) and *y*(*t*), Pearson correlation coefficient is defined in the time domain as:


$$R^{2}=\underset{\tau }{\max}\frac{cov^{2}(x(t),y(t+\tau ))}{var(x(t))\cdot var(y(t+\tau ))}$$

where var, cov and τ denote, respectively, variance, covariance and time-shift between the two time series.^36^ Correlation values are contained between 0 (*y* is uncorrelated with *x*) and 1 (*x* and *y* are fully linearly dependent). The estimation of this parameter is performed on a temporal window of fixed duration and sliding in time in order to follow the temporal evolution of the linear statistical relationship between both signals.^37^ The method investigates also the effect of a shifting temporally the two signals and retains the maximal *R*^2^ value across all explored time-shift. In the present study the following parameters for *R*^2^ analysis were used: time window 4 s, maximum time-shift 0.1 s, steps 1 s. The FC analysis was conducted on a grey matter bipolar montage, excluding white matter channels. When constructing the connectivity matrix, the values that referred to the connection of intra-thalamic channels were removed to highlight the connections between the thalamus and the cortical areas. For each thalamic bipolar channel, we first average *R*^2^ values through the time windows. Then the node strength was computed by summing the *R*^2^ values between the thalamic bipolar channel and all the other non-thalamic bipolar channels and normalizing by the number of non-thalamic bipolar channels. In order to obtain one single thalamic strength value for patient's recording (one for rest and one for sleep), the mean between the node strength of the available thalamic channels was calculated, which is referred to as ‘thalamic strength’. The analysis was firstly conducted on all thalamic channels and later considering pulvinar channels only, obtaining similar results. The analysis was realized on broadband signal, but also on raw signals filtered in classically defined EEG sub-bands: delta (0.5–3.4 Hz), theta (3.4–7.4 Hz), alpha (7.4–12.4 Hz), beta (12.4–24 Hz) and gamma (24–80 Hz).^6^

Analyses of the FC results were also investigated according to the epileptogenicity of the structure. Epileptogenicity was determined using the Epileptogenic Index^38^ (EI) that is a semi-automatic method to quantify the ictal discharges.^39^ Briefly, EI estimates the propensity of a brain region to generate fast discharge early during the seizure course. Channels were considered in the EZ if the EI > 0.4, in the propagation zone (PZ) if EI included between 0.1 and 0.4 and in the non-involved zone (NIZ) if EI < 0.1.

As for interictal activities, for 10 patients we assessed the intra-patient reliability of the *R*^2^ measures by repeating the computation on a second sleep recording and checking for significant differences with the previous measures.

### Statistical analyses

A multivariate analysis to evaluate the correlation between clinical characteristics (age, duration of epilepsy and surgical outcome) was performed.

To evaluate the relationships between the paroxysmal interictal activities recorded in the thalamus (spikes and HFO) and the thalamic FC measure (thalamic strength) and patient's surgical outcome, primary objectives of this study, the Spearman rank correlation was used. Because of the low number of patients being Engel IV after surgery (11 patients, but 9 who received thermocoagulations), ANOVA with the Wilcoxon test was performed (after finding a non-normal distribution of the variables) considering only two possible measures of outcome, differentiated by the persistence of invalidating seizures (Engel III and IV versus Engel II and I), as already done in a previous study by our group.^13^ We then repeated the analyses grouping according to seizure freedom after surgery (Engel II, III and IV versus Engel I). After that, we repeated the analyses only on patients who had received thermocoagulations during the SEEG and not subsequent surgery. To test the validity of our results on a more homogeneous measure of outcome, we repeated the analyses using the Engel class score 12 months after SEEG for patients who did not undergo surgery (but who underwent thermocoagulation during SEEG) and the Engel class score 12 months after surgery for those who underwent surgery after SEEG. Additionally, the association of interictal activities and thalamic strength with the clinical variables was studied. Similarly, for continuous variables such as ‘duration of epilepsy’ and ‘age at SEEG’ the Spearman rank correlation was performed; for categorical variables such as ‘aetiology’, ‘epilepsy type’ and ‘lateralization’, ANOVA with the Wilcoxon test was used. The significance for multiple comparisons was corrected using the False Discovery Rate method.

To assess the intra-patient reliability of the measures (spike rate, *R*^2^), we used a Wilcoxon signed-rank test to see if the differences between the means of the values from the sleep recording used for the main analyses and the mean of the values from the second sleep recording were significant.

Finally, the correlation between Engel class and duration of epilepsy was calculated, and, in the same way, the correlation between Engel class and age at the moment of SEEG, using the Spearman rank correlation test. Data were judged to be statistically significant when *P* < 0.05. The statistical analyses were performed using the software R.

### Standard protocol approvals, registrations and patient consents

Written patient consent was obtained both for the implantation of the electrodes for SEEG and for the subsequent radiofrequency-thermocoagulation procedures; the study has received approval from the review board (PADS24-45_dgr).

## Results

### Characteristics of patients

One hundred twenty-one patients (59 females) were analysed in this study (the clinical features are detailed in Table 1).

**Table 1:** Clinical characteristics of the cohort

Sex (female/male)		59/62	Thalamus involved in EZ (EI > 0.4), *n* (%)		21 (17.3%)
Median age at epilepsy onset, years (min-max)		12 (0–47)	Thalamus implantation, *n*°	Pulvinar	88
Median age at SEEG, years (min-max)		28 (3–70)		Others thalamic nuclei	33
Median epilepsy duration, years (min-max)		12 (1–51)	Median follow-up post SEEG, months (min-max)		18 (2–120)
EZ lateralization, right/left/bilateral		53/51/17	Non-pharmacological treatment received, *n*° (%)	Thermocoagulations	97 (80.1%)
Epilepsy type, *n*°(%)	Frontal	8 (6.6%)		Surgery	48 (39.7%)
	Insulo-opercular	11 (9%)		Stimulations (VNS, DBS and/or tDCS)	26 (21.5%)
	Occipital	2 (1.6%)	Engel class, *n*° (%)	I	47 (38.8%)
	Parietal	6 (5%)		II	30 (25%)
	Temporal	48 (39.8%)		III	24 (19.8%)
	Multi-lobar	45 (37.2%)		IV	11 (9%)
	Not defined	1 (0.8%)		No follow-up	9 (7.4%)

SEEG, stereotactic-EEG; EZ, epileptogenic zone; VNS, vagus nerve stimulation; DBS, deep brain stimulation; tDCS, transcranial direct current stimulation.

Correlations of both age and duration of epilepsy with surgical outcome were found to be statistically not significant.

### Hyperexcitability of the thalamus

**Figure 1:**
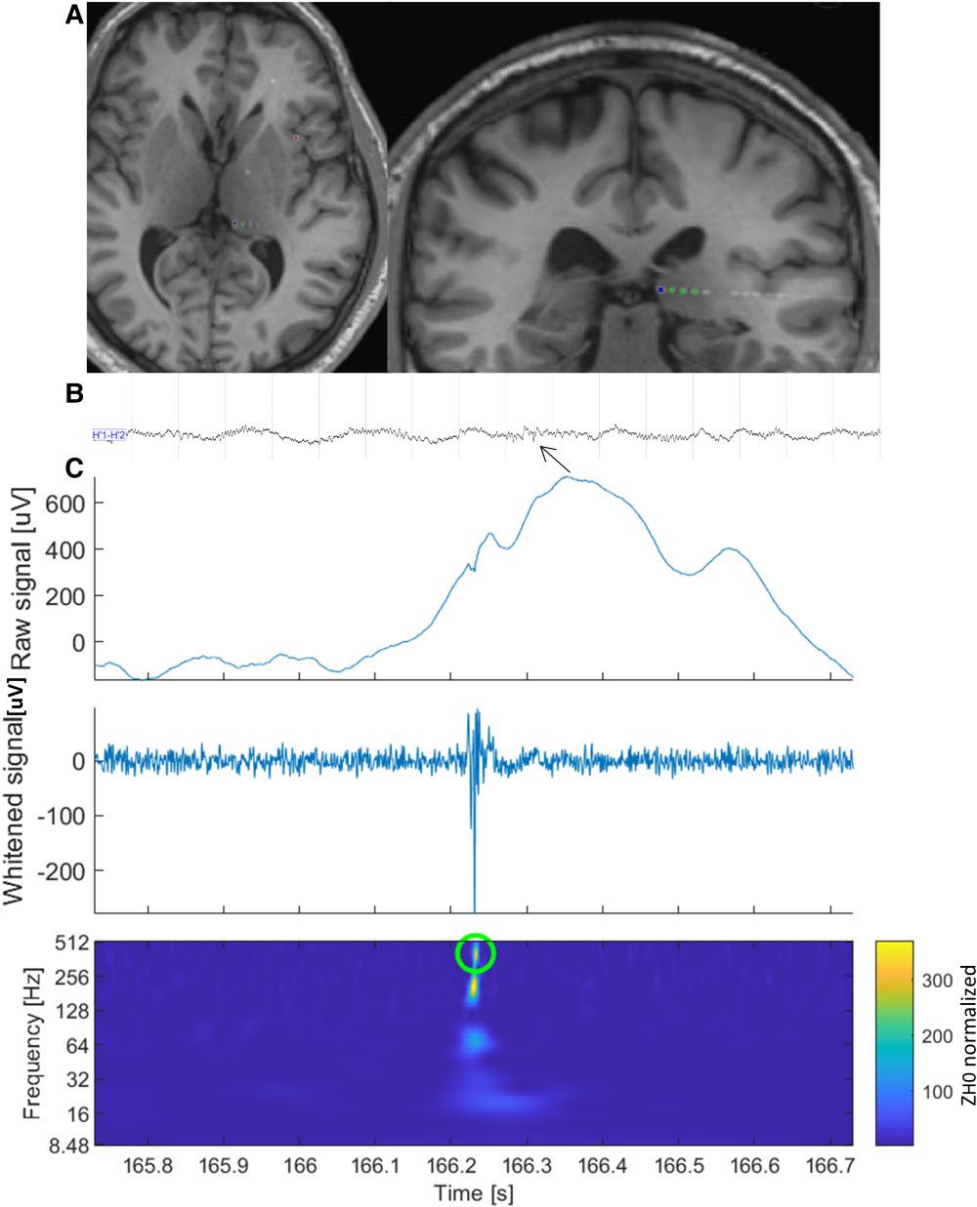
**Thalamic implantation and signal recordings.** (**A**) MRI and CT co-registration showing a SEEG electrode (dotted line) whose distal contacts (green and blue dots) are implanted at the level of the left pulvinar. (**B**) A SEEG signal was recorded at the level of the two distal contacts of the electrode implanted in the pulvinar, in a sleep recording, recognizable for slow wave and spindle activity. The presence of interictal epileptiform activity, a spike, is observed (arrow). (**C**) Raw signal, whitened signal and time-frequency visualization of a Fast Ripple in the pulvinar (green circle).^34^ CT, computed tomography.

To investigate the level of thalamic hyperexcitability during interictal periods, we examined the rates of interictal paroxysmal biomarkers. Specifically, we focused on two key biomarkers: spikes (as illustrated in Fig. 1) and HFO,^40^ with a specific emphasis on fast ripples, defined as oscillations occurring within the frequency range of 250–500 Hz.^41^ Controversies on ripples (80–250 Hz) about their physiological role make this marker less appropriate for epileptogenicity, so we choose to analyse only fast ripples, which are less sensitive but more specific for the EZ, to allow a better interpretation of our results. An automatic detector was employed to systematically analyse and quantify these biomarkers.^34^ No significant differences in the spike rates were found when the intra-patient reliability of the measures was checked on a second set of sleep recordings for 10 patients (reported in the Supplementary material).

**Figure 2:**
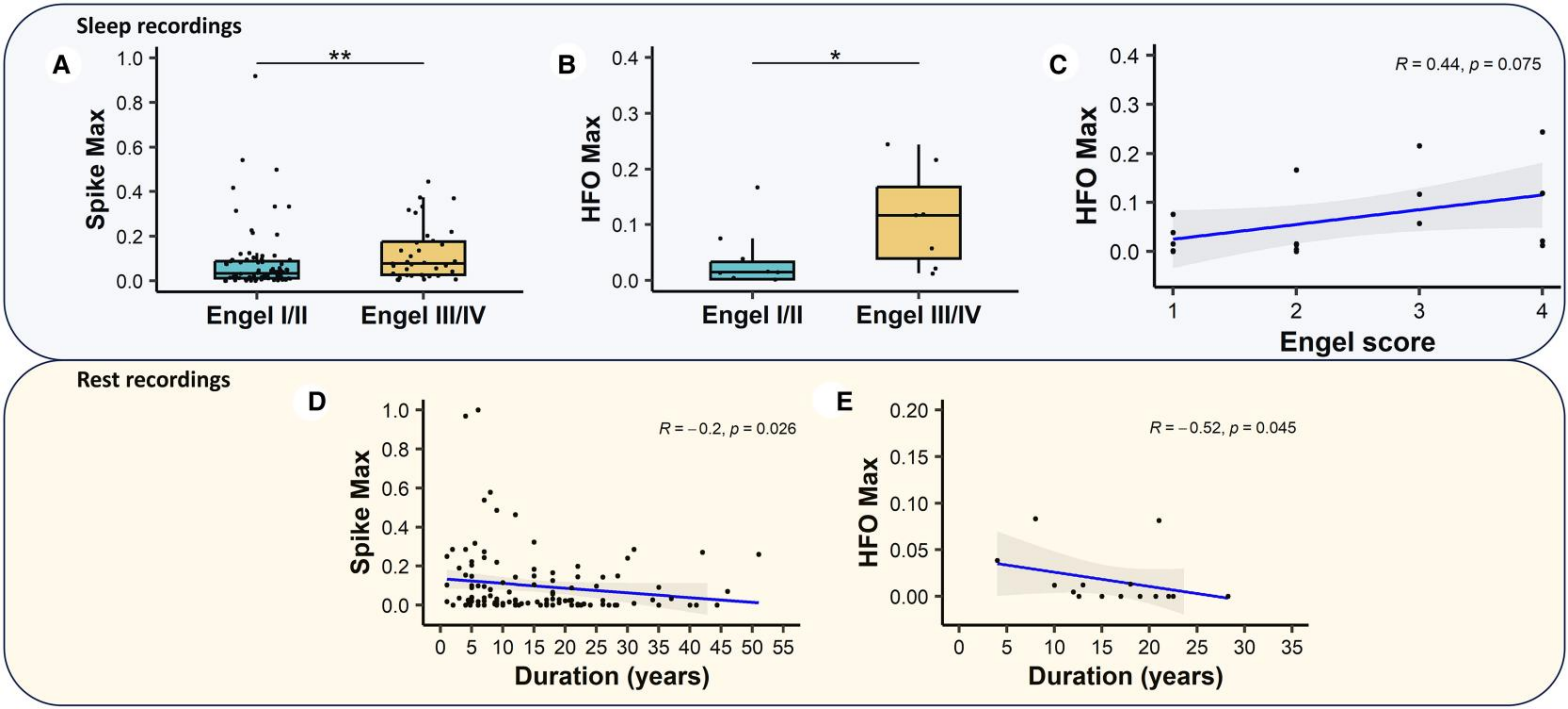
**Thalamic interictal biomarkers associated with hyperexcitability and clinical characteristics.** (**A**) Association between surgical outcome and spikes [*N* = 117, mean estimate = −0.032, 95% CI (−0.065; −0.006), Wilcoxon test: *P* = 0.009, effect size = 0.25] and (**B**) HFO [*N* = 17, mean estimate = −0.075, 95% CI (−0.200; −0.006), Wilcoxon test: *P* = 0.036, effect size = 0.52] in sleep recordings. (**C**) HFO rate according to Engel score [*N* = 17, *r* = 0.44, 95% CI (−0.07; 0.77), *P* = 0.075]. (**D**) Association between epilepsy duration and spikes [*N* = 121, *r* = −0.20, 95% CI (−0.37; −0.022), *P* = 0.026) and (**E**) HFOs (*N* = 15, *r* = −0.52, 95% CI (−0.83; 0.02), *P* = 0.045] in rest recordings.

The investigation aimed to establish a correlation between hyperexcitability biomarker rates and surgical outcomes, as determined by the Engel score.^31^ We found a higher thalamic spike rate during sleep in patients with bad surgical outcomes (Engel Class III/IV) compared to those with good outcomes (Engel Class I/II) [*N* = 117, mean estimate = −0.032, 95% CI (−0.065; −0.006), Wilcoxon test: *P* = 0.009, effect size = 0.25, Fig. 2A]. Additionally, the rate of thalamic HFO, specifically fast ripples (250–500 Hz), during sleep was also higher in patients with bad surgical outcome compared to those with good outcome [*N* = 17, mean estimate = −0.075, 95% CI (−0.200; −0.006), Wilcoxon test: *P* = 0.036, effect size = 0.52, Fig. 2 B]. We also observed a slight increasing trend in the HFO rate with the severity of Engel classes [*r*(17)= 0.44, 95% CI (−0.07; 0.77), *P* = 0.075; Fig. 2C]. Importantly, these associations were observed only during sleep recordings and not during rest. The group-level findings suggest that thalamic hyperexcitability during sleep, as measured by interictal paroxysmal biomarkers, may serve as a potential new marker for predicting epilepsy outcomes after surgery. It is remarkable that the effect size and mean difference obtained from the analysis of the relationship between outcome and HFO are higher than those obtained from the measurement of spikes.

During rest recordings, we observed a reduction of interictal paroxysmal activities, both in terms of spike and HFO rates [*r*(121)= −0.20, 95% CI (−0.37; −0.022), *P* = 0.026 for spikes and *r*(15)= −0.52, 95% CI (−0.83; 0.02), *P* = 0.045 for HFO as depicted in Fig. 2 D and Fig. 2 E], in patients with longer duration of epilepsy. This suggests a potential relationship between thalamic hyperexcitability and the temporal evolution of epilepsy. We also checked the possible correlation between Engel class and duration of epilepsy, and, in the same way, the correlation between Engel class and age at the moment of SEEG, without finding any significance.

When the analyses were repeated only in patients who had undergone thermocoagulation during SEEG and not subsequent surgery, we obtained similar results, losing significance for fast ripples (shown in the Supplementary material).

Repeating the analyses using the Engel class score 12 months after SEEG for patients who did not undergo surgery (but who underwent thermocoagulation during SEEG) and the Engel class score 12 months after surgery for those who underwent surgery after SEEG, we obtained similar results, except for spikes in sleep recordings where significance was lost (as reported in the Supplementary material).

No differences were found in Spike and HFO rates concerning various factors such as age at SEEG, epilepsy type, lateralization, aetiology and thalamic involvement in the EZ (defined by EI values > 0.4 in the thalamic channels),^38^ or when grouping patients according to seizure freedom at follow-up (Engel II, III and IV versus Engel I) (analyses included in Supplementary material).

### Thalamo-cortical FC

To examine the functional relationships between the thalamus and the cortical regions, particularly considering their roles in the ictal epileptogenic network (defined within EZ, PZ or NIZ),^1^ a FC analysis was conducted using linear correlation coefficient (*R*^2^). As for the interictal activities, no significant differences in the *R*^2^ values were found when the intra-patient reliability of the measures was checked on a second set of sleep recordings for 10 patients (reported in the Supplementary material).

**Figure 3:**
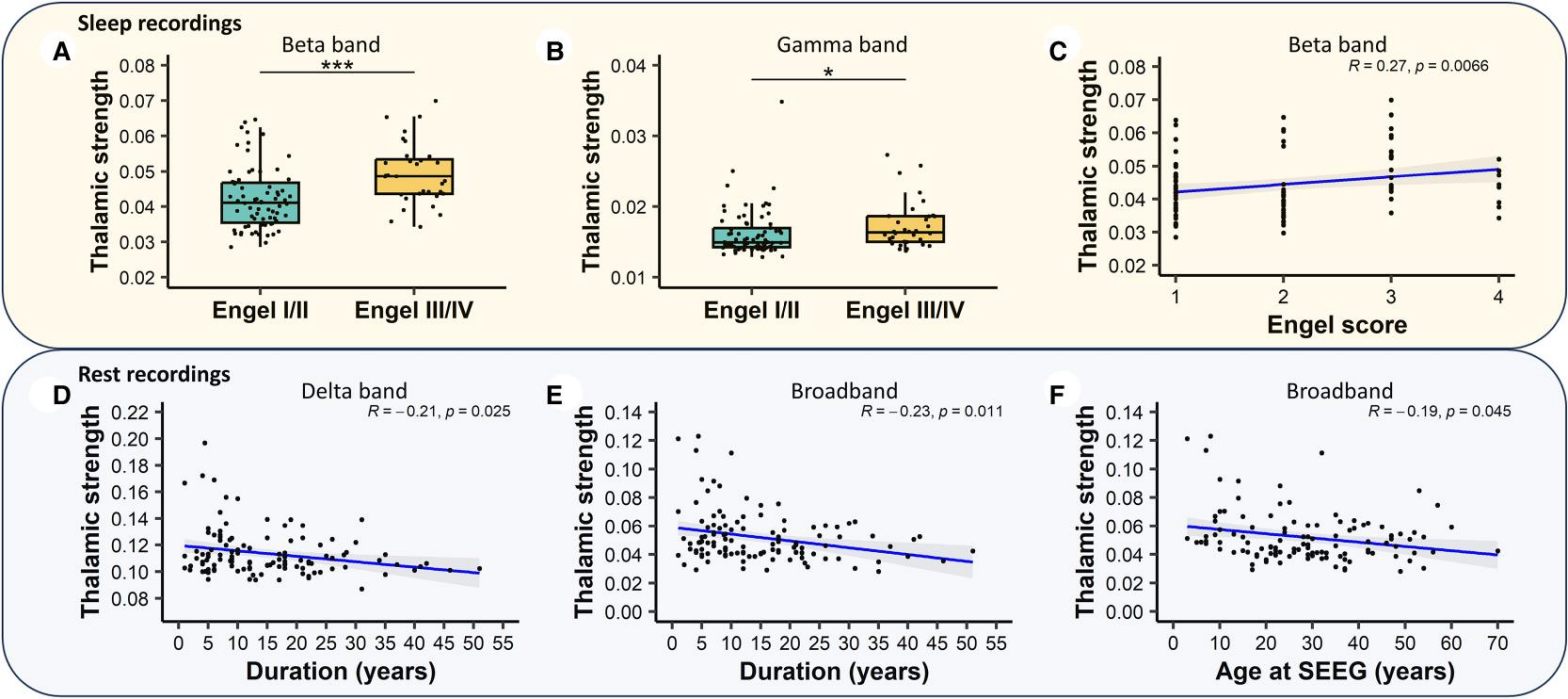
**Thalamo-cortical interictal FC and clinical characteristics.** (**A**) Association between surgical outcome and FC in beta (*N* = 115, mean estimate = −0.007, 95% CI (−0.011; −0.004), Wilcoxon test: *P* < 0.001, effect size = 0.38) and (**B**) gamma band [*N* = 116, mean estimate = −0.001, 95% CI (−0.002; −0.0001), Wilcoxon test: *P* = 0.012, effect size = 0.24] in sleep recordings. (**C**) FC in beta band varies according to Engel score [*N* = 115, *r* = 0.267, 95% CI (0.073; 0.441), *P* = 0.007]. (**D**) Association between epilepsy duration and thalamo-cortical FC in delta band [*N* = 120, *r* = −0.208, 95% CI (−0.377; −0.024), *P* = 0.025] and (**E**) broadband [*N* = 120, *r* = −0.234, 95% CI (−0.401; −0.052), *P* = 0.01] in rest recordings. (**F**) Association between age at SEEG and thalamo-cortical FC in broadband [*N* = 120, *r* = −0.186, 95% CI (−0.357; −0.003), *P* = 0.045] in rest recordings.

Analysis of thalamic strength during sleep recordings showed higher values for patients with bad post-surgical outcomes (Engel Class III/IV) compared to those with a good one (Engel Class I/II). Notably, these differences were observed in the beta and gamma bands [*N* = 115, mean estimate = −0.007, 95% CI (−0.011; −0.004), Wilcoxon test: *P* < 0.001, effect size = 0.38 for beta band and *N* = 116, mean estimate = −0.001, 95% CI (−0.002; −0.0001), Wilcoxon test: *P* = 0.012, effect size = 0.24 for gamma band; depicted in Fig. 3 A and B], but not in broadband and other sub-bands (delta, theta, alpha). Furthermore, in the beta band, a discernible increasing trend in thalamic strength values with the severity of Engel classes was noted [*r*(115)= 0.267, 95% CI (0.073; 0.441), *P* = 0.007, Fig. 3C]. Repeating the analyses using the Engel class score 12 months after SEEG for patients who did not undergo surgery and the Engel class score 12 months after surgery for those who underwent surgery after SEEG, we obtained similar results, except for gamma band in sleep recordings (as reported in the Supplementary material).

It has been further explored if thalamic FC was different according to the sub-networks (EZ, PZ, NIZ). We found higher connectivity with the NIZ in sleep recordings in the beta and gamma bands [*N* = 115, mean estimate = −0.008, 95% CI (−0.011; −0.004), Wilcoxon test: *P* < 0.001, effect size = 0.41 for beta band and *N* = 116, mean estimate = −0.001, 95% CI (−0.002; −0.0004), Wilcoxon test: *P* = 0.003, effect size = 0.28 for gamma band] in patients with higher Engel class. Therefore, connections within the non-involved networks emerged as more influential on the thalamo-cortical correlation with surgical outcomes. Importantly, none of these correlations with surgical outcomes were identified during rest recordings.

In addition, thalamic strength values reduced with the duration of epilepsy during rest recordings, in both delta band and broadband [*r*(120) = −0.208, 95% CI (−0.377; −0.024), *P* = 0.025 for delta band and *r*(120)= −0.234, 95% CI (−0.401; −0.052), *P* = 0.01 for broadband; Fig. 3 D and E]. A similar thalamic strength value reduction was also identified for older patients (higher age at SEEG) during rest recordings in broadband [*r*(120)= −0.186, 95% CI (−0.357; −0.003), *P* = 0.045; Fig. 3F]. No differences for thalamic strength were found regarding aetiology, epilepsy type and lateralization or when grouping patients according to seizure freedom at follow-up (Engel II, III and IV versus Engel I) (analyses included in Supplementary material).

Regarding the calculated effect sizes, it appears that the analysis of thalamic strength in the beta band may be more relevant, whereas the mean difference and the effect size between thalamic strength in the gamma band of patients with good and poor outcomes are lower in our cohort.

When examining thalamic FC broadband in rest recordings according to the sub-networks (EZ, PZ and NIZ), we found a reduction following the duration of epilepsy [*r*(120)= −0.247, 95% CI (−0.412; −0.066), *P* = 0.007) and age at SEEG (*r*(120)= −0.193, 95% CI (−0.363; −0.010), *P* = 0.037] were specifically evident with the NIZ, while did not emerge in the involved zone, obtained merging the PZ and the EZ.

Also for FC, there were no differences in results when analyses were performed only on patients who received thermocoagulation during SEEG and not subsequent surgery (analyses included in Supplementary material).

## Discussion

In this study, findings from a large cohort of 121 patients with thalamic SEEG recordings are presented, marking the largest cohort to date. This group-level exploration of interictal SEEG recordings of the thalamus, mainly explored in the pulvinar, shows associations with post-surgical epilepsy severity outcomes, but also contributes with the broader understanding of subcortical-cortical interactions in focal epilepsies.

A notable aspect of the study lies in the differentiation of thalamic properties based on the patient's vigilance state—awake or during NREM sleep. These states provide distinct information offering insights into surgical outcomes (sleep) and into the disease course and evolution (awake rest). This suggests that the thalamus harbours critical information for both the progression of the disease and the ability to predict surgical outcomes. Thus, these observations reinforce the role of the thalamus in focal epilepsy, supporting the modulation by DBS approaches for influencing epileptogenic network dynamics.

Analysis of FC revealed informative patterns, particularly in thalamic connections with cortical areas outside the epileptogenic network. This complexity underscores the intricate organization of focal epilepsies and potential explanations for varied neurocognitive and neuropsychological comorbidities. Building on prior work investigating cortical FC in the interictal state,^6^ where the non-involved network played a crucial role in predicting surgical outcomes, current findings reaffirm the broad and nuanced nature of what is conventionally labelled as “focal” epilepsy.

### Thalamic hyperexcitability biomarkers and FC during sleep are linked to the surgical outcome

This study revealed a compelling association between hyperexcitability biomarkers (higher rate of spikes and HFO) in the thalamus during sleep recordings and unfavourable surgical outcomes. Few previous studies reported the presence of hyperexcitability biomarkers in the thalamus, mainly spikes^10^ and in a small cohort of patients.^20^ In this last study,^20^ the presence of thalamic spikes was associated with a worse DBS outcome. These results are consistent with the notion that the extension of the spike network could be a prognosis factor in patients operated after SEEG.^42^ However, it is probably an index of the extension of the epileptogenicity, having in mind that surgical outcome after SEEG is more related to ictal than interictal biomarkers.^43^

Concerning HFO in thalamus, a scarcity of data in human studies prompted this investigation.^21^ Although HFO detection was limited to a small number of patients (rest *N* = 15, sleep *N* = 17) due to sampling rate constraints, this result provides valuable insights into the potential significance of thalamic HFO as outcome predictor, underscoring the need for further exploration.

This investigation, employing the *R*^2^ linear correlation method to explore FC, also reveals an association of higher values of FC in the thalamus, particularly during sleep recordings in the beta and gamma bands, and worse surgical outcomes. This observation aligns seamlessly with neuroimaging studies, where patients experiencing persistent seizures post-temporal lobe epilepsy surgery exhibited notable white matter abnormalities in thalamo-temporal tracts,^44^ disrupted right thalamo-hippocampus resting state FC,^45^ and a specific increase in nodal hubness involving both ipsilateral and contralateral thalami.^46^ Our results add a new dimension to this understanding by demonstrating that the thalamo-cortical FC on SEEG, particularly with the NIZ, plays a pivotal role in predicting surgical outcomes. This observation is in line with previous works showing relationship between altered FC extent and surgical outcome.^47^

Notably, hyperexcitability biomarkers in the mesial temporal region are profoundly shaped by vigilance states^48^ prompting their exploration within the thalamus. Recent studies suggest NREM sleep as the best vigilance state to identify the EZ network^49,50^ establishing it as a critical period for prognostic insights. The impact of spikes during sleep^51^ extends beyond the nocturnal realm, influencing neurological performance during wakefulness.^52^ The findings of this study, aligned with previous research on neocortical regions, establish correlations between hyperexcitability biomarkers and surgical prognosis in the thalamus, specifically during sleep.^53^

In summary, the findings of this study underscore the role of thalamic hyperexcitability biomarkers and thalamo-cortical FC during sleep in predicting surgical outcomes in focal drug-resistant epilepsy. This insight, derived from neurophysiological studies, enriches the understanding of the dynamics governing the efficacy of surgical interventions in epilepsy.

### Thalamic hyperexcitability biomarkers and FC decrease with epilepsy duration and age during rest recordings

During resting-state recordings, a distinctive correlation between the duration of epilepsy and changes in thalamic interictal paroxysmal activity was found, suggesting a reduction in FC over time. Specifically, a progressive reduction was observed in hyperexcitability biomarkers measured in the thalamus, potentially linked to a concurrent reduction in FC with increasing epilepsy duration.

An inverse relationship between the duration of epilepsy and EEG FC has been demonstrated in other studies on intracranial^54^ and surface neurophysiological recordings.^55^ Changes in SEEG network topology in relation to epilepsy duration have also been demonstrated in the past.^56^ Moreover, several MRI studies have shown variations in white matter volume^57^ and abnormal fractional anisotropy (between the homolateral insula and the superior temporal pole)^58^ associated with the duration of epilepsy. Longer durations of epilepsy have also been associated with lower subcortical volume, including thalamus, and cortical thickness.^59^ This research confirms possible effects of epilepsy duration on FC involving the thalamus.

In parallel, age-related differences in thalamo-cortical connectivity were found, a topic less explored in the context of epilepsy. The existing literature suggests that brain's functional and structural connectivity patterns decrease with older age in normal subjects^60^ and in a more pronounced way in patients with Alzheimer disease, in particular in the default mode network.^61^ Focusing on the thalamus, age-related differences in thalamo-cortical connectivity may contribute with age-related changes in attention, working memory and episodic memory processes.^62^

The SEEG recordings also revealed a lower thalamo-cortical FC in older patients, independent of epilepsy duration. This reduction during resting state recordings hints at a potential parallel with the well-documented functional-MRI-default network modification with age.

### Methodological aspects and considerations

The strength of this study is the semi-automation of the signal analysis process. Indeed, after rest and sleep recordings selection according to the criteria described in the methods section, and exclusion of bad channels and artefacts, the analysis of interictal activity and FC were performed automatically on all patients with an open-source software (http://meg.univ-amu.fr/wiki/AnyWave) promoting reproducibility.

One relevant limitation of this study is that the analyses were performed by grouping patients according to their surgical outcome, without conducting analyses at the individual level to determine whether truly critical thresholds of values could be identified and how they perform in predicting the surgical outcome in individual patients. Therefore, future analyses to demonstrate the applicability of our results in clinical practice are necessary and will be the subject of further investigations.

A limitation of our study is the use of the Engel class score at the last available follow-up, which introduced variability in the timing of outcome assessments between patients and may have reduced the homogeneity of the results. To address this, we repeated the analyses using the Engel class score 12 months after SEEG for patients who did not undergo surgery (but who underwent thermocoagulation during SEEG) and the Engel class score 12 months after surgery for those who underwent surgery after SEEG (reported in the Supplementary material). While most of the results were similar, the Wilcoxon signed-rank tests for spike and gamma band FC are not significant in the second analyses (still with *P*-values equal or close to 0.1). The numerosity of the sample and its distribution in the Engel classes remain factors that may influence the robustness and generalizability of the results. About the lack of significant results in the Engel I versus II-IV analyses, also this may be related to the distribution of Engel class in our cohort, or may reflect a stronger relationship between our measures and epilepsy severity compared to seizure freedom after outcomes. Future studies with larger and more homogeneous data sets could further clarify these associations and validate the main findings of the study.

Concerning the surgical outcome, one of the major limitations of our study is certainly that we considered patients who underwent conventional surgery and patients who underwent thermocoagulations during SEEG in the same way. This choice was tied to the conceptual similarity between surgery and thermocoagulation regarding the potential role of the thalamus as subcortical hub. However, to confirm the results obtained, we repeated the analysis only in patients who had undergone thermocoagulation during SEEG and not subsequent surgery, and found similar results except for fast ripples, probably due to the small sample size for this second analysis (*N* = 10). On the other hand, it was not possible to repeat the analysis considering patients who had received only conventional surgery, because in our case series there are only two patients (2 pts Engel III, 0 pts Engel IV) who continued to have disabling seizures after surgery, preventing a statistical comparison with seizure-free patients after surgery (32 pts Engel I, 11 pts Engel II).

Another possible bias in this study is related to the anti-seizure medications that the patients were taking during their hospitalization to record the SEEG. Usually, anti-seizure medications were withdrawn during the SEEG recording to facilitate seizures. Therefore, considering that we selected two SEEG recordings (rest and NREM) for each patient, it is possible that the recordings for each patient were influenced by different anti-seizure medications or concentrations of these drugs.

Considering the NREM recordings analysed, a possible limitation of this study could be related to the fact that we selected the recordings according to the characteristics of the intracranial recordings only (slow waves and spindles in frontal neocortical regions and in the thalamus), without the usual polysomnographic setting.

Our results come from a cohort of patients with forms of focal epilepsies in whom exploration of the *planum temporale* and/or Heschl's gyrus was required for clinical reasons. In fact, most of these patients have only one thalamic electrode implanted in the posterior part of the thalamus. This limits us to an analysis of the neurophysiological activities of the pulvinar and the posterior part of the thalamus. In fact, a more detailed subdivision of the thalamic subnuclei and a more specific evoked potential study of the thalamo-cortical connections are lacking. Further analysis in this sense will be useful to better understand specific cortico-subthalamic relationships, especially to possibly improve DBS target selection. The results of this study are applicable to types of epilepsy involving implantation in the *planum temporale* and/or Heschl's gyrus. Consequently, they may not be generalizable to other types of focal epilepsies, such as prefrontal lobe epilepsy, which may have different characteristics.

## Conclusions

This study illustrates innovative findings regarding thalamic SEEG exploration. The thalamus exhibits heightened interictal activity (both spikes and HFO) and FC during sleep in patients with poor surgical outcomes compared to those with favourable outcomes after surgery and/or thermocoagulations. Certainly, more extensive data collection, specifically focused on interictal thalamic activity, and analyses to investigate the reproducibility of our results to a patient level are paramount before the effective application of our results to a clinical population. Nevertheless, the integration of these initial and exploratory results with the clinical and quantitative data obtained from the pre-surgical evaluation could help to identify patients who are not suitable for surgery and possibly guide them towards alternative clinical interventions, such as DBS.

Additionally, this study highlights a reduction in interictal activity and FC within the thalamus associated with epilepsy duration and age in rest recordings. This prompts speculation about the implications of persistent epilepsy on brain networks and the possible role of the thalamus as a central hub of connections in the ageing process.

In conclusion, the thalamus provides information differently depending on the vigilance state, with more epilepsy-related insights unearthed during slow-wave sleep. Conversely, during the resting state, thalamic neurophysiology appears more linked to the ‘default mode network’, the ageing of patients and epilepsy evolution.

Further analyses, incorporating both resting and sleep SEEG recordings, are essential to uncover specific connections between the thalamus and distinct brain areas. This detailed exploration will contribute with identifying targets for tailoring stimulation protocols, offering a more personalized approach to each patient.

## Supplementary Material

fcaf102_Supplementary_Data

## Data Availability

The authors confirm that the data supporting the findings of this study and the code used for the analyses are available within the article and/or its Supplementary material.
