# Supplementary material for: Interictal waking and sleep electrophysiological properties of the thalamus in focal epilepsies
Source: Brain Commun. 2025 Mar 5;7(2):fcaf102. doi: 10.1093/braincomms/fcaf102 (PMC11934066; doi:10.1093/braincomms/fcaf102)
Supplement: fcaf102_Supplementary_Data [file fcaf102_supplementary_data.pdf]

Supplementary Material

Additional Analyses

Intracohort reproducibility (Wilcoxon signed-rank test for Spike, HFO and thalamic strength measures) studied on 10 patients

Spike rate

```
        Wilcoxon signed rank test with continuity correction

data:  data$max_spike_rate_thalamus_sleep and data$`2 max_spike_rate_thalamus_sleep`
V = 34.5, p-value = 0.5073
alternative hypothesis: true location shift is not equal to 0
```

HFO rate

```
        Wilcoxon signed rank test with continuity correction

data:  data$max_HFO_rate_thalamus_sleep and data$`2 max_HFO_rate_thalamus_sleep`
V = 1, p-value = 1
alternative hypothesis: true location shift is not equal to 0
```

R^2 Thalamic electrodes VS all non-thalamic electrodes

```
        Wilcoxon signed rank exact test

data:  data$thVSnonth_broadband_r2_sleep_mean and data$`2 thVSnonth_broadband_r2_sleep_mean`
V = 23, p-value = 0.6953
alternative hypothesis: true location shift is not equal to 0

        Wilcoxon signed rank exact test

data:  data$thVSnonth_alpha_r2_sleep_mean and data$`2 thVSnonth_alpha_r2_sleep_mean`
V = 24, p-value = 0.9102
alternative hypothesis: true location shift is not equal to 0

        Wilcoxon signed rank exact test

data:  data$thVSnonth_beta_r2_sleep_mean and data$`2 thVSnonth_beta_r2_sleep_mean`
V = 25, p-value = 0.8203
alternative hypothesis: true location shift is not equal to 0

        Wilcoxon signed rank exact test

data:  data$thVSnonth_delta_r2_sleep_mean and data$`2 thVSnonth_delta_r2_sleep_mean`
V = 24, p-value = 0.9102
alternative hypothesis: true location shift is not equal to 0

        Wilcoxon signed rank exact test

data:  data$thVSnonth_gamma_r2_sleep_mean and data$`2 thVSnonth_gamma_r2_sleep_mean`
V = 33, p-value = 0.25
alternative hypothesis: true location shift is not equal to 0
```

```
        Wilcoxon signed rank exact test

data:  data$thVSnonth_theta_r2_sleep_mean and data$`2 thVSnonth_theta_r2_sleep_mean`
V = 29, p-value = 0.4961
alternative hypothesis: true location shift is not equal to 0
```

R^2 Thalamic electrodes VS electrodes in the Propagation Zone

```
        Wilcoxon signed rank exact test

data:  data$thVSPZ_broadband_r2_sleep_mean and data$`2 thVSPZ_broadband_r2_sleep_mean`
V = 27, p-value = 1
alternative hypothesis: true location shift is not equal to 0

        Wilcoxon signed rank exact test

data:  data$thVSPZ_alpha_r2_sleep_mean and data$`2 thVSPZ_alpha_r2_sleep_mean`
V = 23, p-value = 1
alternative hypothesis: true location shift is not equal to 0

        Wilcoxon signed rank exact test

data:  data$thVSPZ_beta_r2_sleep_mean and data$`2 thVSPZ_beta_r2_sleep_mean`
V = 27, p-value = 0.6523
alternative hypothesis: true location shift is not equal to 0

        Wilcoxon signed rank exact test

data:  data$thVSPZ_delta_r2_sleep_mean and data$`2 thVSPZ_delta_r2_sleep_mean`
V = 23, p-value = 1
alternative hypothesis: true location shift is not equal to 0

        Wilcoxon signed rank exact test

data:  data$thVSPZ_gamma_r2_sleep_mean and data$`2 thVSPZ_gamma_r2_sleep_mean`
V = 32, p-value = 0.3008
alternative hypothesis: true location shift is not equal to 0

        Wilcoxon signed rank exact test

data:  data$thVSPZ_theta_r2_sleep_mean and data$`2 thVSPZ_theta_r2_sleep_mean`
V = 31, p-value = 0.3594
alternative hypothesis: true location shift is not equal to 0
```

R^2 Thalamic electrodes VS electrodes in the Non-Involved Zone

```
        Wilcoxon signed rank exact test

data:  data$thVSnonPZ_broadband_r2_sleep_mean and data$`2 thVSnonPZ_broadband_r2_sleep_mean`
V = 18, p-value = 0.375
alternative hypothesis: true location shift is not equal to 0
```

```
        Wilcoxon signed rank exact test

data:  data$thVsnonPZ_alpha_r2_sleep_mean and data$`2 thVsnonPZ_alpha_r2_sleep_mean`
V = 24, p-value = 0.9102
alternative hypothesis: true location shift is not equal to 0

        Wilcoxon signed rank exact test

data:  data$thVsnonPZ_beta_r2_sleep_mean and data$`2 thVsnonPZ_beta_r2_sleep_mean`
V = 23, p-value = 1
alternative hypothesis: true location shift is not equal to 0

        Wilcoxon signed rank exact test

data:  data$thVsnonPZ_delta_r2_sleep_mean and data$`2 thVsnonPZ_delta_r2_sleep_mean`
V = 18, p-value = 0.6523
alternative hypothesis: true location shift is not equal to 0

        Wilcoxon signed rank exact test

data:  data$thVsnonPZ_gamma_r2_sleep_mean and data$`2 thVsnonPZ_gamma_r2_sleep_mean`
V = 31, p-value = 0.3594
alternative hypothesis: true location shift is not equal to 0

        Wilcoxon signed rank exact test

data:  data$thVsnonPZ_theta_r2_sleep_mean and data$`2 thVsnonPZ_theta_r2_sleep_mean`
V = 28, p-value = 0.5703
alternative hypothesis: true location shift is not equal to 0
```

Analyses for re-grouped surgical outcome at last FU (Engel I vs Engel II-IV)

Hyperexcitability of the thalamus

---Wilcoxon test for thalamic spike rate in sleep recordings: not significant; N= 117, mean estimate=

-0.007, 95% CI [-0.028; 0.008] p = 0.299, effect size = 0.098

---Wilcoxon test for thalamic HFO rate in sleep recordings: not significant; N= 17, mean estimate=

-0.020, 95% CI [-0.165; 0.023] p = 0.268, effect size = 0.282

Thalamo-cortical functional connectivity

---Wilcoxon test for thalamic strength beta-band in sleep recordings: not significant; N= 115, mean estimate= -0.003, 95% CI [-0.007; 0.001] p = 0.098, effect size = 0.164

---Wilcoxon test for thalamic strength gamma-band in sleep recordings: not significant; N= 116, mean estimate= -0.0001, 95% CI = [-0.001; 0.001] p = 0.744, effect size = 0.032

Analyses for thermocoagulations-only pts evaluated at last FU (Engel I-II vs Engel III-IV)

WILCOXON SIGNED RANK TEST

- Association between surgical outcome (Engel 1-2 vs Engel 3-4) and spikes for sleep records (N= 56, W = 284, p= 0.034)

- Association between surgical outcome (Engel 1-2 vs Engel 3-4) and HFOs for sleep records (N= 10, W = 6, p= 0.16)

- Association between surgical outcome (Engel 1-2 vs Engel 3-4) and FC in beta (N= 55, W = 258, p = 0.018)

- Association between surgical outcome (Engel 1-2 vs Engel 3-4) and FC in gamma (N= 56, W = 237, p = 0.007)

Analyses for surgical outcome evaluated at 1 year after thermocoagulation= or surgery (Engel I-II vs Engel III-IV)

WILCOXON SIGNED RANK TEST (PAIRED DATA)

data: data$Engel_1_year and data$Engel_last_FU, V = 98.5, p-value = 0.56

CORRELATIONS

- HFO rate according to Engel score at 12 months for sleep records (Spearman's rank correlation: N= 17, r= 0.34, p= 0.18).

- FC in beta band according to Engel score for sleep records (Spearman's rank correlation: N= 102, r= 0.19, p= 0.05).

WILCOXON SIGNED RANK TEST

- Association between surgical outcome (Engel 1-2 vs Engel 3-4) and spikes for sleep records (N= 104, W = 995, p= 0.12)

- Association between surgical outcome (Engel 1-2 vs Engel 3-4) and HFOs for sleep records (N= 17, W = 15.5, p= 0.05)

- Association between surgical outcome (Engel 1-2 vs Engel 3-4) and FC in beta for sleep records (N= 102, W = 779, p = 0.004)

- Association between surgical outcome (Engel 1-2 vs Engel 3-4) and FC in gamma for sleep records (N= 103, W = 974, p = 0.10)

Post-hoc Power Analysis

To assess the potential influence of sample size on our findings, we conducted a post-hoc power analysis at $\alpha = 0.05$ for some of the results presented in the paper. The results are as follows:

Spike Rate in Sleep: Power = 32%

HFO Rate in Sleep: Power = 61%

Beta-band functional connectivity in sleep: Power = 93.9%

Gamma-band functional connectivity in sleep: Power = 34.2%

These findings suggest that our study was adequately powered to detect effects in the Beta-band functional connectivity analysis (93.9%), but may have been underpowered for Spike Rate (32%) and Gamma-band connectivity (34.2%), meaning that the lack of statistical significance in these analyses could be partially attributed to the limited sample size rather than the absence of an effect, and this might have influenced the additional analyses performed.

For reference, the Engel classification at the last available follow-up was:

-Spike rate and functional connectivity analyses: 71 patients (Engel I-II), 34 patients (Engel III-IV)

-HFO rate analysis: 10 patients (Engel I-II), 7 patients (Engel III-IV)

```
##################################################################################
#                                                    #
#                    Script R BrainCom                        #
#                     20/10/2024 (EG)          Engel I/II vs Engel III-IV              #
#                                                    #
##################################################################################


rm(list=ls())

## packages ##
library(readxl)
library(dplyr)
library(ggplot2)
library(reshape2)
library(ggpubr)
library(rstatix)
library(paletteer)

## Function to compute 95% confidence intervals for Spearman correlation ##
spearman_CI <- function(x, y, alpha = 0.05){
  rs <- cor(x, y, method = "spearman", use = "complete.obs")
  n <- sum(complete.cases(x, y))
  CI <- sort(tanh(atanh(rs) + c(-1,1)*sqrt((1+rs^2/2)/(n-3))*qnorm(p = alpha/2)))
  return(list("R" = rs, "df" = n-2, "CI" = CI))
}

## Path ##
path_data <- "E:/Thalamus_Tommaso/1.13.1.Data/Data_paper" # Set your data path
```

```
############################ SPIKE and HFO ANALYSIS ############################

## Load data ##

data_rest <- read_excel(paste(path_data, "2023.05.17.delphos_rest_norm.xlsx",
                        sep = "/"))
head(data_rest)
data_sleep <- read_excel(paste(path_data, "2023.05.17.delphos_sleep_norm.xlsx",
                         sep = "/"))
head(data_sleep)
data_clinic <- read_excel(paste(path_data, "2023.05.17.clinical_data.xlsx",
                          sep = "/"))
colnames(data_clinic)[1] <- "sub"
head(data_clinic)
data_clinic$sub <- gsub('sub-','', data_clinic$sub)


## Type of variable ##
data_clinic$duration <- as.numeric(data_clinic$duration)


## List of patients ##
patients <- unique(data_clinic$sub)


## Merge clinical and analytic data ##
data_rest <- data_rest[data_rest$sub %in% patients,]
data_rest <- left_join(data_rest, data_clinic, by = "sub")
data_sleep <- data_sleep[data_sleep$sub %in% patients,]
data_sleep <- left_join(data_sleep, data_clinic, by = "sub")


## Select thalamic contacts ##
data_rest$thalamus <- rep("no", nrow(data_rest))
data_sleep$thalamus <- rep("no", nrow(data_sleep))
for (pat in 1:length(patients)) {
  tha_contacts <- unlist(strsplit(data_clinic$electrode[data_clinic$sub == patients[pat]], ";"))
  data_rest$thalamus[data_rest$sub == patients[pat] & data_rest$Channel %in% tha_contacts] <- "yes"
  data_sleep$thalamus[data_sleep$sub == patients[pat] & data_sleep$Channel %in% tha_contacts] <- "yes"
}


## Re-order data ##
data_rest <- data_rest[data_rest$thalamus == "yes",
                 c(1, 5, 37, 6:9, 35, 17, 19:21, 24, 26, 28)]
data_sleep <- data_sleep[data_sleep$thalamus == "yes",
```

```
               c(1, 5, 37, 6:9, 35, 17, 19:21, 24, 26, 28)]

## Select HFO data ##
data_rest_FR_tmp <- data_rest[data_rest$SEEG_rest_freq == 2048, ]
data_sleep_FR_tmp <- data_sleep[data_sleep$SEEG_sleep_freq == 2048, ]

## Compute max Spike rate and HFO per patient ##
# Spike #
data_rest_SR <- unique(data_rest[,c(1,8:15)])
data_rest_SR <- merge(data_rest_SR,
                 aggregate(. ~ sub, data_rest[,c(1,4)], max),
                 by = "sub")
colnames(data_rest_SR)[10] <- c("Spike_max")

data_sleep_SR <- unique(data_sleep[,c(1,8:15)])
data_sleep_SR <- merge(data_sleep_SR,
                  aggregate(. ~ sub, data_sleep[,c(1,4)], max),
                  by = "sub")
colnames(data_sleep_SR)[10] <- c("Spike_max")

# HFO #
data_rest_FR <- unique(data_rest_FR_tmp[,c(1,8:15)])
data_rest_FR <- merge(data_rest_FR,
                 aggregate(. ~ sub, data_rest_FR_tmp[,c(1,5)], max),
                 by = "sub")
colnames(data_rest_FR)[10] <- c("Fast_Ripple_max")

data_sleep_FR <- unique(data_sleep_FR_tmp[,c(1,8:15)])
data_sleep_FR <- merge(data_sleep_FR,
                  aggregate(. ~ sub, data_sleep_FR_tmp[,c(1,5)], max),
                  by = "sub")
colnames(data_sleep_FR)[10] <- c("Fast_Ripple_max")

## Add Engel score labels ##
# Spike #
data_rest_SR$SF <- rep(NA, nrow(data_rest_SR))
data_rest_SR$SF[data_rest_SR$engel_class_code %in% c("1", "2")] <- "Engel I/II"
data_rest_SR$SF[data_rest_SR$engel_class_code %in% c("3", "4")] <- "Engel III/IV"
data_rest_SR$SF <- factor(data_rest_SR$SF,
```

```
                    levels = c("Engel I/II", "Engel III/IV"))
data_sleep_SR$SF <- rep(NA, nrow(data_sleep_SR))
data_sleep_SR$SF[data_sleep_SR$engel_class_code %in% c("1", "2")] <- "Engel I/II"
data_sleep_SR$SF[data_sleep_SR$engel_class_code %in% c("3", "4")] <- "Engel III/IV"
data_sleep_SR$SF <- factor(data_sleep_SR$SF,
                     levels = c("Engel I/II", "Engel III/IV"))

# HFO #
data_rest_FR$SF <- rep(NA, nrow(data_rest_FR))
data_rest_FR$SF[data_rest_FR$engel_class_code %in% c("1", "2")] <- "Engel I/II"
data_rest_FR$SF[data_rest_FR$engel_class_code %in% c("3", "4")] <- "Engel III/IV"
data_rest_FR$SF <- factor(data_rest_FR$SF,
                    levels = c("Engel I/II", "Engel III/IV"))
data_sleep_FR$SF <- rep(NA, nrow(data_sleep_FR))
data_sleep_FR$SF[data_sleep_FR$engel_class_code %in% c("1", "2")] <- "Engel I/II"
data_sleep_FR$SF[data_sleep_FR$engel_class_code %in% c("3", "4")] <- "Engel III/IV"
data_sleep_FR$SF <- factor(data_sleep_FR$SF,
                     levels = c("Engel I/II", "Engel III/IV"))

## Figure 2 ##
# Figure 2a Spike Rate Max : Engel I/II vs Engel III/IV - Sleep #
pwc <- data_sleep_SR[is.na(data_sleep_SR$SF) == F,] %>%
  pairwise_wilcox_test(Spike_max ~ SF, p.adjust.method = "fdr", detailed = T)
pwc <- pwc %>% add_xy_position(x = "SF")
pwc
data_sleep_SR[is.na(data_sleep_SR$SF) == F,] %>% wilcox_effsize(Spike_max ~ SF)
(p <- ggboxplot(data_sleep_SR[is.na(data_sleep_SR$SF) == F,],
            x = "SF", y = "Spike_max",
            fill = "SF", outlier.shape = NA, width = 0.5, size = 0.5) +
    geom_boxplot(aes(fill = SF), outlier.shape = NA, width = 0.5, size = 0.5,
             fatten = 1, color = "black") +
    geom_point(size = 0.2, position = position_jitter(0.2)) +
    stat_pvalue_manual(pwc, hide.ns = TRUE, label = "p.adj.signif",
                 tip.length = 0) +
    xlab(NULL) + ylab("Spike Max") +
    theme_classic() +
    theme(plot.title = element_text(size = 12, face = "bold", hjust = 0.5)) +
    theme(axis.title = element_text(size = 9, face = "bold"),
        axis.text.x = element_text(colour = "black", size = 9, face = "bold"),
```

```
        axis.text.y = element_text(colour = "black", size = 7)) +
    theme(legend.text = element_text(size = 12),
        legend.title=element_blank(),
        legend.margin = margin(1,1,1,1, unit = "mm"),
        legend.direction = "horizontal",
        legend.position = "none") +
    scale_y_continuous(breaks = seq(0, 1, 0.2), limits = c(0, 1)) +
    scale_fill_paletteer_d("rcartocolor::Pastel"))
png("boxplot_sleep_SpikeMax_engel.png", width = 6, height = 4, units = "cm",
    res = 700)
print(p)
dev.off()


# Figure 2b HFO Max : Engel I/II vs Engel III/IV - Sleep #
pwc <- data_sleep_FR[is.na(data_sleep_FR$SF) == F,] %>%
  pairwise_wilcox_test(Fast_Ripple_max ~ SF, p.adjust.method = "fdr",
                      detailed = T)
pwc <- pwc %>% add_xy_position(x = "SF")
pwc$y.position <- 0.39
pwc
data_sleep_FR[is.na(data_sleep_FR$SF) == F,] %>% wilcox_effsize(Fast_Ripple_max ~ SF)
(p <- ggboxplot(data_sleep_FR[is.na(data_sleep_FR$SF) == F,],
               x = "SF", y = "Fast_Ripple_max",
               fill = "SF", outlier.shape = NA, width = 0.5, size = 0.5) +
    geom_boxplot(aes(fill = SF), outlier.shape = NA, width = 0.5, size = 0.5,
                fatten = 1, color = "black") +
    geom_point(size = 0.2, position = position_jitter(0.2)) +
    stat_pvalue_manual(pwc, hide.ns = TRUE, label = "p.adj.signif",
                      tip.length = 0) +
    xlab(NULL) + ylab("HFO Max") +
    theme_classic() +
    theme(plot.title = element_text(size = 12, face = "bold", hjust = 0.5)) +
    theme(axis.title = element_text(size = 9, face = "bold"),
        axis.text.x = element_text(colour = "black", size = 9, face = "bold"),
        axis.text.y = element_text(colour = "black", size = 7)) +
    theme(legend.text = element_text(size = 12),
        legend.title=element_blank(),
        legend.margin = margin(1,1,1,1, unit = "mm"),
        legend.direction = "horizontal",
```

```
        legend.position = "none") +
    scale_y_continuous(breaks = seq(0, 1, 0.1), limits = c(0, 0.4)) +
    scale_fill_paletteer_d("rcartocolor::Pastel"))
png("boxplot_sleep_HFOMax_engel.png", width = 6, height = 4, units = "cm",
    res = 700)
print(p)
dev.off()

# Figure 2c HFO Max vs Duration - Sleep #
data_sleep_FR$engel_class_code <- as.numeric(data_sleep_FR$engel_class_code)
spearman_CI(data_sleep_FR$Fast_Ripple_max, data_sleep_FR$engel_class_code)
(p <- ggscatter(data = data_sleep_FR,
            x = "engel_class_code", y = "Fast_Ripple_max",
            size = 0, add = "reg.line", conf.int = T,
            add.params = list(color = "blue", fill = "lightgray",
                          size = 0.5)) +
    geom_point(size = 0.3) +
    stat_cor(method = "spearman", label.x = 2.8, label.y = 0.38, size = 2)  +
    xlab("Engel score") + ylab("HFO Max") +
    theme_classic() +
    theme(plot.title = element_text(size = 12, face = "bold", hjust = 0.5)) +
    theme(axis.title = element_text(size = 9, face = "bold"),
        axis.text = element_text(colour = "black", size = 7)) +
    theme(legend.text = element_text(size = 12),
        legend.title=element_blank(),
        legend.margin = margin(1,1,1,1, unit = "mm"),
        legend.direction = "horizontal",
        legend.position = "bottom") +
    scale_x_continuous(breaks = seq(1, 4, 1), limits = c(1, 4)) +
    scale_y_continuous(breaks = seq(-0.1, 1, 0.1), limits = c(-0.05, 0.4)))
png("corr_sleep_HFOMax_engel.png", width = 6, height = 4, units = "cm",
    res = 700)
print(p)
dev.off()

# Figure 2d Spike rate Max vs Duration - Rest #
spearman_CI(data_rest_SR$Spike_max, data_rest_SR$duration)
(p <- ggscatter(data = data_rest_SR,
            x = "duration", y = "Spike_max",
```

```
            size = 0, add = "reg.line", conf.int = T,
            add.params = list(color = "blue", fill = "lightgray",
                              size = 0.5)) +
  geom_point(size = 0.3) +
  stat_cor(method = "spearman", label.x = 35, label.y = 0.9, size = 2)  +
  xlab("Duration (years)") + ylab("Spike Max") +
  theme_classic() +
  theme(plot.title = element_text(size = 12, face = "bold", hjust = 0.5)) +
  theme(axis.title = element_text(size = 9, face = "bold"),
        axis.text = element_text(colour = "black", size = 7)) +
  theme(legend.text = element_text(size = 10),
        legend.title=element_blank(),
        legend.margin = margin(1,1,1,1, unit = "mm"),
        legend.direction = "horizontal",
        legend.position = "bottom") +
  scale_x_continuous(breaks = seq(0, 55, 5), limits = c(0, 55)) +
  scale_y_continuous(breaks = seq(0, 1, 0.2), limits = c(-0.05, 1)))
png("corr_rest_spikeMax_duration.png", width = 6, height = 4, units = "cm",
  res = 700)
print(p)
dev.off()

# Figure 2e HFO Max vs Duration - Rest #
spearman_CI(data_rest_FR$Fast_Ripple_max, data_rest_FR$duration)
(p <- ggscatter(data = data_rest_FR,
            x = "duration", y = "Fast_Ripple_max",
            size = 0, add = "reg.line", conf.int = T,
            add.params = list(color = "blue", fill = "lightgray",
                              size = 0.5)) +
  geom_point(size = 0.3) +
  stat_cor(method = "spearman", label.x = 20, label.y = 0.18, size = 2)  +
  xlab("Duration (years)") + ylab("HFO Max") +
  theme_classic() +
  theme(plot.title = element_text(size = 12, face = "bold", hjust = 0.5)) +
  theme(axis.title = element_text(size = 9, face = "bold"),
        axis.text = element_text(colour = "black", size = 7)) +
  theme(legend.text = element_text(size = 12),
        legend.title=element_blank(),
        legend.margin = margin(1,1,1,1, unit = "mm"),
```

```
        legend.direction = "horizontal",
        legend.position = "bottom") +
    scale_x_continuous(breaks = seq(0, 55, 5), limits = c(0, 35)) +
    scale_y_continuous(breaks = seq(-0.1, 1, 0.05), limits = c(-0.02, 0.2)))
png("corr_rest_HFOMax_duration.png", width = 6, height = 4, units = "cm",
    res = 700)
print(p)
dev.off()



############################ CONNECTIVITY ANALYSIS #############################
## Load data ##
data_FC_broadband <- read_excel(paste(path_data,
                                "2023.11.06.r2_thVSnonth_broadband.xlsx",
                                sep = "/"))
colnames(data_FC_broadband)[1] <- "sub"
data_FC_broadband$sub <- gsub('sub-','', data_FC_broadband$sub)
data_FC_broadband <- data_FC_broadband[complete.cases(data_FC_broadband),]
head(data_FC_broadband)
data_FC_beta <- read_excel(paste(path_data,
                                "2023.11.06.r2_thVSnonth_beta.xlsx",
                                sep = "/"))
colnames(data_FC_beta)[1] <- "sub"
data_FC_beta$sub <- gsub('sub-','', data_FC_beta$sub)
data_FC_beta <- data_FC_beta[complete.cases(data_FC_beta),]
head(data_FC_beta)
data_FC_gamma <- read_excel(paste(path_data,
                             "2023.11.06.r2_thVSnonth_gamma.xlsx",
                             sep = "/"))
colnames(data_FC_gamma)[1] <- "sub"
data_FC_gamma$sub <- gsub('sub-','', data_FC_gamma$sub)
data_FC_gamma <- data_FC_gamma[complete.cases(data_FC_gamma),]
head(data_FC_gamma)
data_FC_delta <- read_excel(paste(path_data,
                             "2023.11.06.r2_thVSnonth_delta.xlsx",
                             sep = "/"))
colnames(data_FC_delta)[1] <- "sub"
data_FC_delta$sub <- gsub('sub-','', data_FC_delta$sub)
```

```
data_FC_delta <- data_FC_delta[complete.cases(data_FC_delta),]
head(data_FC_delta)

## Merge clinical and FC data ##
data_broadband <- left_join(data_clinic[, c(1,18,20,29)],
                            data_FC_broadband[, c(1,2,4)],
                    by = "sub")
colnames(data_broadband)[5:6] <- c("FC_rest_broadband", "FC_sleep_broadband")

data_beta <- left_join(data_clinic[, c(1,18,20,29)],
                       data_FC_beta[, c(1,2,4)],
                             by = "sub")
colnames(data_beta)[5:6] <- c("FC_rest_beta", "FC_sleep_beta")

data_gamma <- left_join(data_clinic[, c(1,18,20,29)],
                        data_FC_gamma[, c(1,2,4)],
                          by = "sub")
colnames(data_gamma)[5:6] <- c("FC_rest_gamma", "FC_sleep_gamma")

data_delta <- left_join(data_clinic[, c(1,18,20,29)],
                           data_FC_delta[, c(1,2,4)],
                           by = "sub")
colnames(data_delta)[5:6] <- c("FC_rest_delta", "FC_sleep_delta")

## Figure 3 ##
# Figure 3a FC Beta band : Engel I/II vs Engel III/IV - Sleep #
## Add Engel score labels ##
data_beta$SF <- rep(NA, nrow(data_beta))
data_beta$SF[data_beta$engel_class_code %in% c("1", "2")] <- "Engel I/II"
data_beta$SF[data_beta$engel_class_code %in% c("3", "4")] <- "Engel III/IV"
data_beta$SF <- factor(data_beta$SF,
                    levels = c("Engel I/II", "Engel III/IV"))
pwc <- data_beta[is.na(data_beta$SF) == F,] %>%
  pairwise_wilcox_test(FC_sleep_beta ~ SF, p.adjust.method = "fdr", detailed = T)
pwc <- pwc %>% add_xy_position(x = "SF")
pwc$y.position <- 0.078
pwc
data_beta[is.na(data_beta$SF) == F,] %>% wilcox_effsize(FC_sleep_beta ~ SF)
(p <- ggboxplot(data_beta[is.na(data_beta$SF) == F,],
```

```
                x = "SF", y = "FC_sleep_beta",
                fill = "SF", outlier.shape = NA, width = 0.5, size = 0.5) +
    geom_boxplot(aes(fill = SF), outlier.shape = NA, width = 0.5, size = 0.5, fatten = 1, color = "black") +
    geom_point(size = 0.2, position = position_jitter(0.2)) +
    stat_pvalue_manual(pwc, hide.ns = TRUE, label = "p.adj.signif", tip.length = 0) +

    xlab(NULL) + ylab("Thalamic strength") +
    theme_classic() +
    theme(plot.title = element_text(size = 12, face = "bold", hjust = 0.5)) +
    theme(axis.title = element_text(size = 9, face = "bold"),
          axis.text.x = element_text(colour = "black", size = 9, face = "bold"),
          axis.text.y = element_text(colour = "black", size = 7)) +
    theme(legend.text = element_text(size = 12),
          legend.title=element_blank(),
          legend.margin = margin(1,1,1,1, unit = "mm"),
          legend.direction = "horizontal",
          legend.position = "none") +
    scale_y_continuous(breaks = seq(0, 0.1, 0.01), limits = c(0.02, 0.08)) +
    scale_fill_paletteer_d("rcartocolor::Pastel"))
png("boxplot_beta_sleep_R2Mean_engel.png", width = 6, height = 4, units = "cm",
    res = 700)
print(p)
dev.off()

# Figure 3b FC Gamma band : Engel I/II vs Engel III/IV - Sleep #
## Add Engel score labels ##
data_gamma$SF <- rep(NA, nrow(data_gamma))
data_gamma$SF[data_gamma$engel_class_code %in% c("1", "2")] <- "Engel I/II"
data_gamma$SF[data_gamma$engel_class_code %in% c("3", "4")] <- "Engel III/IV"
data_gamma$SF <- factor(data_gamma$SF,
                        levels = c("Engel I/II", "Engel III/IV"))
pwc <- data_gamma[is.na(data_gamma$SF) == F,] %>%
  pairwise_wilcox_test(FC_sleep_gamma ~ SF, p.adjust.method = "fdr", detailed = T)
pwc <- pwc %>% add_xy_position(x = "SF")
pwc$y.position <- 0.038
pwc
data_gamma[is.na(data_gamma$SF) == F,] %>% wilcox_effsize(FC_sleep_gamma ~ SF)
(p <- ggboxplot(data_gamma[is.na(data_gamma$SF) == F,],
                x = "SF", y = "FC_sleep_gamma",
```

```
            fill = "SF", outlier.shape = NA, width = 0.5, size = 0.5) +
  geom_boxplot(aes(fill = SF), outlier.shape = NA, width = 0.5, size = 0.5, fatten = 1, color = "black") +
  geom_point(size = 0.2, position = position_jitter(0.2)) +
  stat_pvalue_manual(pwc, hide.ns = TRUE, label = "p.adj.signif", tip.length = 0) +

  xlab(NULL) + ylab("Thalamic strength") +
  theme_classic() +
  theme(plot.title = element_text(size = 12, face = "bold", hjust = 0.5)) +
  theme(axis.title = element_text(size = 9, face = "bold"),
        axis.text.x = element_text(colour = "black", size = 9, face = "bold"),
        axis.text.y = element_text(colour = "black", size = 7)) +
  theme(legend.text = element_text(size = 12),
        legend.title=element_blank(),
        legend.margin = margin(1,1,1,1, unit = "mm"),
        legend.direction = "horizontal",
        legend.position = "none") +
  scale_y_continuous(breaks = seq(0, 0.1, 0.01), limits = c(0.01, 0.04)) +
  scale_fill_paletteer_d("rcartocolor::Pastel"))
png("boxplot_gamma_sleep_R2Mean_engel.png", width = 6, height = 4, units = "cm",
  res = 700)
print(p)
dev.off()

# Figure 3c FC beta band vs engel score - Sleep #
data_beta$engel_class_code <- as.numeric(data_beta$engel_class_code)
spearman_CI(data_beta$FC_sleep_beta, data_beta$engel_class_code)
(p <- ggscatter(data = data_beta,
           x = "engel_class_code", y = "FC_sleep_beta",
           size = 0, add = "reg.line", conf.int = T,
           add.params = list(color = "blue", fill = "lightgray",
                        size = 0.5)) +
 geom_point(size = 0.3) +
 stat_cor(method = "spearman", label.x = 2.6, label.y = 0.08, size = 2)  +
 xlab("Engel score") + ylab("Thalamic strength") +
 theme_classic() +
 theme(plot.title = element_text(size = 12, face = "bold", hjust = 0.5)) +
 theme(axis.title = element_text(size = 9, face = "bold"),
       axis.text = element_text(colour = "black", size = 7)) +
 theme(legend.text = element_text(size = 12),
```

```
        legend.title=element_blank(),
        legend.margin = margin(1,1,1,1, unit = "mm"),
        legend.direction = "horizontal",
        legend.position = "bottom") +
  scale_x_continuous(breaks = seq(1, 4, 1), limits = c(1, 4)) +
  scale_y_continuous(breaks = seq(0, 0.08, 0.01), limits = c(0.02, 0.08)))
png("corr_beta_sleep_R2Mean_engel.png", width = 6, height = 4, units = "cm",
    res = 700)
print(p)
dev.off()

# Figure 3d FC delta band vs duration - Rest #
data_delta$duration <- as.numeric(data_delta$duration)
spearman_CI(data_delta$FC_rest_delta, data_delta$duration)
(p <- ggscatter(data = data_delta,
                x = "duration", y = "FC_rest_delta",
                size = 0, add = "reg.line", conf.int = T,
                add.params = list(color = "blue", fill = "lightgray", size = 0.5)) +
  geom_point(size = 0.3) +
  stat_cor(method = "spearman", label.x = 32, label.y = 0.22, size = 2)  +
  xlab("Duration (years)") + ylab("Thalamic strength") +
  theme_classic() +
  theme(plot.title = element_text(size = 12, face = "bold", hjust = 0.5)) +
  theme(axis.title = element_text(size = 9, face = "bold"),
        axis.text = element_text(colour = "black", size = 7)) +
  theme(legend.text = element_text(size = 12),
        legend.title=element_blank(),
        legend.margin = margin(1,1,1,1, unit = "mm"),
        legend.direction = "horizontal",
        legend.position = "bottom") +
  scale_x_continuous(breaks = seq(0, 55, 5), limits = c(0, 55))  +
  scale_y_continuous(breaks = seq(0, 0.22, 0.02), limits = c(0.08, 0.22)))
png("corr_delta_rest_R2Mean_duration.png", width = 6, height = 4, units = "cm",
    res = 700)
print(p)

# Figure 3e FC broadband vs duration - Rest #
data_broadband$duration <- as.numeric(data_broadband$duration)
spearman_CI(data_broadband$FC_rest_broadband, data_broadband$duration)
```

```
(p <- ggscatter(data = data_broadband,
             x = "duration", y = "FC_rest_broadband",
             size = 0, add = "reg.line", conf.int = T,
             add.params = list(color = "blue", fill = "lightgray", size = 0.5)) +
   geom_point(size = 0.3) +
   stat_cor(method = "spearman", label.x = 32, label.y = 0.14, size = 2)  +
   xlab("Duration (years)") + ylab("Thalamic strength") +
   theme_classic() +
   theme(plot.title = element_text(size = 12, face = "bold", hjust = 0.5)) +
   theme(axis.title = element_text(size = 9, face = "bold"),
        axis.text = element_text(colour = "black", size = 7)) +
   theme(legend.text = element_text(size = 12),
        legend.title=element_blank(),
        legend.margin = margin(1,1,1,1, unit = "mm"),
        legend.direction = "horizontal",
        legend.position = "bottom") +
   scale_x_continuous(breaks = seq(0, 55, 5), limits = c(0, 55))  +
   scale_y_continuous(breaks = seq(0, 0.22, 0.02), limits = c(0.0, 0.14)))
png("corr_broadband_rest_R2Mean_duration.png", width = 6, height = 4, units = "cm",
   res = 700)
print(p)

# Figure 3f FC broadband vs age at SEEG - Rest #
data_broadband$age_at_SEEG <- as.numeric(data_broadband$age_at_SEEG)
spearman_CI(data_broadband$FC_rest_broadband, data_broadband$age_at_SEEG)
(p <- ggscatter(data = data_broadband,
            x = "age_at_SEEG", y = "FC_rest_broadband",
            size = 0, add = "reg.line", conf.int = T,
            add.params = list(color = "blue", fill = "lightgray", size = 0.5)) +
  geom_point(size = 0.3) +
  stat_cor(method = "spearman", label.x = 42, label.y = 0.14, size = 2)  + # Add correlation coefficient
  xlab("Age at SEEG (years)") + ylab("Thalamic strength") +
  theme_classic() +
  theme(plot.title = element_text(size = 12, face = "bold", hjust = 0.5)) +
  theme(axis.title = element_text(size = 9, face = "bold"),
       axis.text = element_text(colour = "black", size = 7)) +
  theme(legend.text = element_text(size = 12),
       legend.title=element_blank(),
       legend.margin = margin(1,1,1,1, unit = "mm"),
```

```
        legend.direction = "horizontal",
        legend.position = "bottom") +
  scale_x_continuous(breaks = seq(0, 70, 10), limits = c(0, 70))  +
  scale_y_continuous(breaks = seq(0, 0.14, 0.02), limits = c(0, 0.14)))
png("corr_broadband_rest_R2Mean_age_at_SEEG.png", width = 6, height = 4,
    units = "cm", res = 700)
print(p)
dev.off()

##################################################################################
#                                                          #
#                      Script R BrainCom                         #
#                      20/10/2024 (EG)           Engel I vs Engel II-IV          #
#                                                          #
##################################################################################



rm(list=ls())

## packages ##
library(readxl)
library(dplyr)
library(ggplot2)
library(reshape2)
library(ggpubr)
library(rstatix)
library(paletteer)

## Function to compute 95% confidence intervals for Spearman correlation ##
spearman_CI <- function(x, y, alpha = 0.05){
  rs <- cor(x, y, method = "spearman", use = "complete.obs")
  n <- sum(complete.cases(x, y))
  CI <- sort(tanh(atanh(rs) + c(-1,1)*sqrt((1+rs^2/2)/(n-3))*qnorm(p = alpha/2)))
  return(list("R" = rs, "df" = n-2, "CI" = CI))
}

## Path ##
path_data <- "E:/Thalamus_Tommaso/1.13.1.Data/Data_paper" # Set your data path
```

```
############################ SPIKE and HFO ANALYSIS ############################

## Load data ##
data_rest <- read_excel(paste(path_data, "2023.05.17.delphos_rest_norm.xlsx",
                        sep = "/"))
head(data_rest)
data_sleep <- read_excel(paste(path_data, "2023.05.17.delphos_sleep_norm.xlsx",
                         sep = "/"))
head(data_sleep)
data_clinic <- read_excel(paste(path_data, "2023.05.17.clinical_data.xlsx",
                          sep = "/"))
colnames(data_clinic)[1] <- "sub"
head(data_clinic)
data_clinic$sub <- gsub('sub-','', data_clinic$sub)

## Type of variable ##
data_clinic$duration <- as.numeric(data_clinic$duration)

## List of patients ##
patients <- unique(data_clinic$sub)

## Merge clinical and analytic data ##
data_rest <- data_rest[data_rest$sub %in% patients,]
data_rest <- left_join(data_rest, data_clinic, by = "sub")
data_sleep <- data_sleep[data_sleep$sub %in% patients,]
data_sleep <- left_join(data_sleep, data_clinic, by = "sub")

## Select thalamic contacts ##
data_rest$thalamus <- rep("no", nrow(data_rest))
data_sleep$thalamus <- rep("no", nrow(data_sleep))
for (pat in 1:length(patients)) {
  tha_contacts <- unlist(strsplit(data_clinic$electrode[data_clinic$sub == patients[pat]], ";"))
  data_rest$thalamus[data_rest$sub == patients[pat] & data_rest$Channel %in% tha_contacts] <- "yes"
  data_sleep$thalamus[data_sleep$sub == patients[pat] & data_sleep$Channel %in% tha_contacts] <- "yes"
}

## Re-order data ##
```

```
data_rest <- data_rest[data_rest$thalamus == "yes",
                       c(1, 5, 37, 6:9, 35, 17, 19:21, 24, 26, 28)]
data_sleep <- data_sleep[data_sleep$thalamus == "yes",
                         c(1, 5, 37, 6:9, 35, 17, 19:21, 24, 26, 28)]

## Select HFO data ##
data_rest_FR_tmp <- data_rest[data_rest$SEEG_rest_freq == 2048, ]
data_sleep_FR_tmp <- data_sleep[data_sleep$SEEG_sleep_freq == 2048, ]

## Compute max Spike rate and HFO per patient ##
# Spike #
data_rest_SR <- unique(data_rest[,c(1,8:15)])
data_rest_SR <- merge(data_rest_SR,
                      aggregate(. ~ sub, data_rest[,c(1,4)], max),
                      by = "sub")
colnames(data_rest_SR)[10] <- c("Spike_max")

data_sleep_SR <- unique(data_sleep[,c(1,8:15)])
data_sleep_SR <- merge(data_sleep_SR,
                       aggregate(. ~ sub, data_sleep[,c(1,4)], max),
                       by = "sub")
colnames(data_sleep_SR)[10] <- c("Spike_max")

# HFO #
data_rest_FR <- unique(data_rest_FR_tmp[,c(1,8:15)])
data_rest_FR <- merge(data_rest_FR,
                      aggregate(. ~ sub, data_rest_FR_tmp[,c(1,5)], max),
                      by = "sub")
colnames(data_rest_FR)[10] <- c("Fast_Ripple_max")

data_sleep_FR <- unique(data_sleep_FR_tmp[,c(1,8:15)])
data_sleep_FR <- merge(data_sleep_FR,
                       aggregate(. ~ sub, data_sleep_FR_tmp[,c(1,5)], max),
                       by = "sub")
colnames(data_sleep_FR)[10] <- c("Fast_Ripple_max")

## Add Engel score labels ##
# Spike #
data_rest_SR$SF <- rep(NA, nrow(data_rest_SR))
```

```
data_rest_SR$SF[data_rest_SR$engel_class_code %in% c("1")] <- "Engel I"
data_rest_SR$SF[data_rest_SR$engel_class_code %in% c("2", "3", "4")] <- "Engel II-IV"
data_rest_SR$SF <- factor(data_rest_SR$SF,
                    levels = c("Engel I", "Engel II-IV"))
data_sleep_SR$SF <- rep(NA, nrow(data_sleep_SR))
data_sleep_SR$SF[data_sleep_SR$engel_class_code %in% c("1")] <- "Engel I"
data_sleep_SR$SF[data_sleep_SR$engel_class_code %in% c("2", "3", "4")] <- "Engel II-IV"
data_sleep_SR$SF <- factor(data_sleep_SR$SF,
                     levels = c("Engel I", "Engel II-IV"))

# HFO #
data_rest_FR$SF <- rep(NA, nrow(data_rest_FR))
data_rest_FR$SF[data_rest_FR$engel_class_code %in% c("1")] <- "Engel I"
data_rest_FR$SF[data_rest_FR$engel_class_code %in% c("2", "3", "4")] <- "Engel II-IV"
data_rest_FR$SF <- factor(data_rest_FR$SF,
                    levels = c("Engel I", "Engel II-IV"))
data_sleep_FR$SF <- rep(NA, nrow(data_sleep_FR))
data_sleep_FR$SF[data_sleep_FR$engel_class_code %in% c("1")] <- "Engel I"
data_sleep_FR$SF[data_sleep_FR$engel_class_code %in% c("2", "3", "4")] <- "Engel II-IV"
data_sleep_FR$SF <- factor(data_sleep_FR$SF,
                     levels = c("Engel I", "Engel II-IV"))

## Figure 2 ##
# Figure 2a Spike Rate Max : Engel I vs Engel II-IV - Sleep #
pwc <- data_sleep_SR[is.na(data_sleep_SR$SF) == F,] %>%
  pairwise_wilcox_test(Spike_max ~ SF, p.adjust.method = "fdr", detailed = T)
pwc <- pwc %>% add_xy_position(x = "SF")
pwc
data_sleep_SR[is.na(data_sleep_SR$SF) == F,] %>% wilcox_effsize(Spike_max ~ SF)
(p <- ggboxplot(data_sleep_SR[is.na(data_sleep_SR$SF) == F,],
            x = "SF", y = "Spike_max",
            fill = "SF", outlier.shape = NA, width = 0.5, size = 0.5) +
    geom_boxplot(aes(fill = SF), outlier.shape = NA, width = 0.5, size = 0.5,
             fatten = 1, color = "black") +
    geom_point(size = 0.2, position = position_jitter(0.2)) +
    stat_pvalue_manual(pwc, hide.ns = TRUE, label = "p.adj.signif",
                 tip.length = 0) +
    xlab(NULL) + ylab("Spike Max") +
    theme_classic() +
```

```
  theme(plot.title = element_text(size = 12, face = "bold", hjust = 0.5)) +
  theme(axis.title = element_text(size = 9, face = "bold"),
        axis.text.x = element_text(colour = "black", size = 9, face = "bold"),
        axis.text.y = element_text(colour = "black", size = 7)) +
  theme(legend.text = element_text(size = 12),
        legend.title=element_blank(),
        legend.margin = margin(1,1,1,1, unit = "mm"),
        legend.direction = "horizontal",
        legend.position = "none") +
  scale_y_continuous(breaks = seq(0, 1, 0.2), limits = c(0, 1)) +
  scale_fill_paletteer_d("rcartocolor::Pastel"))
png("boxplot_sleep_SpikeMax_engel.png", width = 6, height = 4, units = "cm",
  res = 700)
print(p)
dev.off()

# Figure 2b HFO Max : Engel I/II vs Engel II-IV - Sleep #
pwc <- data_sleep_FR[is.na(data_sleep_FR$SF) == F,] %>%
  pairwise_wilcox_test(Fast_Ripple_max ~ SF, p.adjust.method = "fdr",
                       detailed = T)
pwc <- pwc %>% add_xy_position(x = "SF")
pwc$y.position <- 0.39
pwc
data_sleep_FR[is.na(data_sleep_FR$SF) == F,] %>% wilcox_effsize(Fast_Ripple_max ~ SF)
(p <- ggboxplot(data_sleep_FR[is.na(data_sleep_FR$SF) == F,],
                x = "SF", y = "Fast_Ripple_max",
                fill = "SF", outlier.shape = NA, width = 0.5, size = 0.5) +
  geom_boxplot(aes(fill = SF), outlier.shape = NA, width = 0.5, size = 0.5,
                fatten = 1, color = "black") +
  geom_point(size = 0.2, position = position_jitter(0.2)) +
  stat_pvalue_manual(pwc, hide.ns = TRUE, label = "p.adj.signif",
                     tip.length = 0) +
  xlab(NULL) + ylab("HFO Max") +
  theme_classic() +
  theme(plot.title = element_text(size = 12, face = "bold", hjust = 0.5)) +
  theme(axis.title = element_text(size = 9, face = "bold"),
        axis.text.x = element_text(colour = "black", size = 9, face = "bold"),
        axis.text.y = element_text(colour = "black", size = 7)) +
  theme(legend.text = element_text(size = 12),
```

```
        legend.title=element_blank(),
        legend.margin = margin(1,1,1,1, unit = "mm"),
        legend.direction = "horizontal",
        legend.position = "none") +
  scale_y_continuous(breaks = seq(0, 1, 0.1), limits = c(0, 0.4)) +
  scale_fill_paletteer_d("rcartocolor::Pastel"))
png("boxplot_sleep_HFOMax_engel.png", width = 6, height = 4, units = "cm",
  res = 700)
print(p)
dev.off()

# Figure 2c HFO Max vs Duration - Sleep #
data_sleep_FR$engel_class_code <- as.numeric(data_sleep_FR$engel_class_code)
spearman_CI(data_sleep_FR$Fast_Ripple_max, data_sleep_FR$engel_class_code)
(p <- ggscatter(data = data_sleep_FR,
          x = "engel_class_code", y = "Fast_Ripple_max",
          size = 0, add = "reg.line", conf.int = T,
          add.params = list(color = "blue", fill = "lightgray",
                    size = 0.5)) +
  geom_point(size = 0.3) +
  stat_cor(method = "spearman", label.x = 2.8, label.y = 0.38, size = 2)  +
  xlab("Engel score") + ylab("HFO Max") +
  theme_classic() +
  theme(plot.title = element_text(size = 12, face = "bold", hjust = 0.5)) +
  theme(axis.title = element_text(size = 9, face = "bold"),
        axis.text = element_text(colour = "black", size = 7)) +
  theme(legend.text = element_text(size = 12),
        legend.title=element_blank(),
        legend.margin = margin(1,1,1,1, unit = "mm"),
        legend.direction = "horizontal",
        legend.position = "bottom") +
  scale_x_continuous(breaks = seq(1, 4, 1), limits = c(1, 4)) +
  scale_y_continuous(breaks = seq(-0.1, 1, 0.1), limits = c(-0.05, 0.4)))
png("corr_sleep_HFOMax_engel.png", width = 6, height = 4, units = "cm",
  res = 700)
print(p)
dev.off()

# Figure 2d Spike rate Max vs Duration - Rest #
```

```
spearman_CI(data_rest_SR$Spike_max, data_rest_SR$duration)
(p <- ggscatter(data = data_rest_SR,
        x = "duration", y = "Spike_max",
        size = 0, add = "reg.line", conf.int = T,
        add.params = list(color = "blue", fill = "lightgray",
                size = 0.5)) +
  geom_point(size = 0.3) +
  stat_cor(method = "spearman", label.x = 35, label.y = 0.9, size = 2)  +
  xlab("Duration (years)") + ylab("Spike Max") +
  theme_classic() +
  theme(plot.title = element_text(size = 12, face = "bold", hjust = 0.5)) +
  theme(axis.title = element_text(size = 9, face = "bold"),
     axis.text = element_text(colour = "black", size = 7)) +
  theme(legend.text = element_text(size = 10),
     legend.title=element_blank(),
     legend.margin = margin(1,1,1,1, unit = "mm"),
     legend.direction = "horizontal",
     legend.position = "bottom") +
  scale_x_continuous(breaks = seq(0, 55, 5), limits = c(0, 55)) +
  scale_y_continuous(breaks = seq(0, 1, 0.2), limits = c(-0.05, 1)))
png("corr_rest_spikeMax_duration.png", width = 6, height = 4, units = "cm",
  res = 700)
print(p)
dev.off()

# Figure 2e HFO Max vs Duration - Rest #
spearman_CI(data_rest_FR$Fast_Ripple_max, data_rest_FR$duration)
(p <- ggscatter(data = data_rest_FR,
        x = "duration", y = "Fast_Ripple_max",
        size = 0, add = "reg.line", conf.int = T,
        add.params = list(color = "blue", fill = "lightgray",
                size = 0.5)) +
  geom_point(size = 0.3) +
  stat_cor(method = "spearman", label.x = 20, label.y = 0.18, size = 2)  +
  xlab("Duration (years)") + ylab("HFO Max") +
  theme_classic() +
  theme(plot.title = element_text(size = 12, face = "bold", hjust = 0.5)) +
  theme(axis.title = element_text(size = 9, face = "bold"),
     axis.text = element_text(colour = "black", size = 7)) +
```

```
  theme(legend.text = element_text(size = 12),
        legend.title=element_blank(),
        legend.margin = margin(1,1,1,1, unit = "mm"),
        legend.direction = "horizontal",
        legend.position = "bottom") +
  scale_x_continuous(breaks = seq(0, 55, 5), limits = c(0, 35)) +
  scale_y_continuous(breaks = seq(-0.1, 1, 0.05), limits = c(-0.02, 0.2)))
png("corr_rest_HFOMax_duration.png", width = 6, height = 4, units = "cm",
  res = 700)
print(p)
dev.off()



############################ CONNECTIVITY ANALYSIS #############################
## Load data ##
data_FC_broadband <- read_excel(paste(path_data,
                                "2023.11.06.r2_thVSnonth_broadband.xlsx",
                                sep = "/"))
colnames(data_FC_broadband)[1] <- "sub"
data_FC_broadband$sub <- gsub('sub-','', data_FC_broadband$sub)
data_FC_broadband <- data_FC_broadband[complete.cases(data_FC_broadband),]
head(data_FC_broadband)
data_FC_beta <- read_excel(paste(path_data,
                                "2023.11.06.r2_thVSnonth_beta.xlsx",
                                sep = "/"))
colnames(data_FC_beta)[1] <- "sub"
data_FC_beta$sub <- gsub('sub-','', data_FC_beta$sub)
data_FC_beta <- data_FC_beta[complete.cases(data_FC_beta),]
head(data_FC_beta)
data_FC_gamma <- read_excel(paste(path_data,
                              "2023.11.06.r2_thVSnonth_gamma.xlsx",
                              sep = "/"))
colnames(data_FC_gamma)[1] <- "sub"
data_FC_gamma$sub <- gsub('sub-','', data_FC_gamma$sub)
data_FC_gamma <- data_FC_gamma[complete.cases(data_FC_gamma),]
head(data_FC_gamma)
data_FC_delta <- read_excel(paste(path_data,
                              "2023.11.06.r2_thVSnonth_delta.xlsx",
```

```
                           sep = "/"))
colnames(data_FC_delta)[1] <- "sub"
data_FC_delta$sub <- gsub('sub-','', data_FC_delta$sub)
data_FC_delta <- data_FC_delta[complete.cases(data_FC_delta),]
head(data_FC_delta)

## Merge clinical and FC data ##
data_broadband <- left_join(data_clinic[, c(1,18,20,29)],
                       data_FC_broadband[, c(1,2,4)],
                by = "sub")
colnames(data_broadband)[5:6] <- c("FC_rest_broadband", "FC_sleep_broadband")

data_beta <- left_join(data_clinic[, c(1,18,20,29)],
                   data_FC_beta[, c(1,2,4)],
                         by = "sub")
colnames(data_beta)[5:6] <- c("FC_rest_beta", "FC_sleep_beta")

data_gamma <- left_join(data_clinic[, c(1,18,20,29)],
                    data_FC_gamma[, c(1,2,4)],
                     by = "sub")
colnames(data_gamma)[5:6] <- c("FC_rest_gamma", "FC_sleep_gamma")

data_delta <- left_join(data_clinic[, c(1,18,20,29)],
                       data_FC_delta[, c(1,2,4)],
                       by = "sub")
colnames(data_delta)[5:6] <- c("FC_rest_delta", "FC_sleep_delta")

## Figure 3 ##
# Figure 3a FC Beta band : Engel I vs Engel II-IV - Sleep #
## Add Engel score labels ##
data_beta$SF <- rep(NA, nrow(data_beta))
data_beta$SF[data_beta$engel_class_code %in% c("1")] <- "Engel I"
data_beta$SF[data_beta$engel_class_code %in% c("2", "3", "4")] <- "Engel II-IV"
data_beta$SF <- factor(data_beta$SF,
                levels = c("Engel I", "Engel II-IV"))
pwc <- data_beta[is.na(data_beta$SF) == F,] %>%
  pairwise_wilcox_test(FC_sleep_beta ~ SF, p.adjust.method = "fdr", detailed = T)
pwc <- pwc %>% add_xy_position(x = "SF")
pwc$y.position <- 0.078
```

```
pwc
data_beta[is.na(data_beta$SF) == F,] %>% wilcox_effsize(FC_sleep_beta ~ SF)
(p <- ggboxplot(data_beta[is.na(data_beta$SF) == F,],
        x = "SF", y = "FC_sleep_beta",
        fill = "SF", outlier.shape = NA, width = 0.5, size = 0.5) +
  geom_boxplot(aes(fill = SF), outlier.shape = NA, width = 0.5, size = 0.5, fatten = 1, color = "black") +
  geom_point(size = 0.2, position = position_jitter(0.2)) +
  stat_pvalue_manual(pwc, hide.ns = TRUE, label = "p.adj.signif", tip.length = 0) +

  xlab(NULL) + ylab("Thalamic strength") +
  theme_classic() +
  theme(plot.title = element_text(size = 12, face = "bold", hjust = 0.5)) +
  theme(axis.title = element_text(size = 9, face = "bold"),
     axis.text.x = element_text(colour = "black", size = 9, face = "bold"),
     axis.text.y = element_text(colour = "black", size = 7)) +
  theme(legend.text = element_text(size = 12),
     legend.title=element_blank(),
     legend.margin = margin(1,1,1,1, unit = "mm"),
     legend.direction = "horizontal",
     legend.position = "none") +
  scale_y_continuous(breaks = seq(0, 0.1, 0.01), limits = c(0.02, 0.08)) +
  scale_fill_paletteer_d("rcartocolor::Pastel"))
png("boxplot_beta_sleep_R2Mean_engel.png", width = 6, height = 4, units = "cm",
  res = 700)
print(p)
dev.off()

# Figure 3b FC Gamma band : Engel I/II vs Engel III/IV - Sleep #
## Add Engel score labels ##
data_gamma$SF <- rep(NA, nrow(data_gamma))
data_gamma$SF[data_gamma$engel_class_code %in% c("1")] <- "Engel I"
data_gamma$SF[data_gamma$engel_class_code %in% c("2", "3", "4")] <- "Engel II-IV"
data_gamma$SF <- factor(data_gamma$SF,
            levels = c("Engel I", "Engel II-IV"))
pwc <- data_gamma[is.na(data_gamma$SF) == F,] %>%
 pairwise_wilcox_test(FC_sleep_gamma ~ SF, p.adjust.method = "fdr", detailed = T)
pwc <- pwc %>% add_xy_position(x = "SF")
pwc$y.position <- 0.038
pwc
```

```
data_gamma[is.na(data_gamma$SF) == F,] %>% wilcox_effsize(FC_sleep_gamma ~ SF)
(p <- ggboxplot(data_gamma[is.na(data_gamma$SF) == F,],
                x = "SF", y = "FC_sleep_gamma",
                fill = "SF", outlier.shape = NA, width = 0.5, size = 0.5) +
   geom_boxplot(aes(fill = SF), outlier.shape = NA, width = 0.5, size = 0.5, fatten = 1, color = "black") +
   geom_point(size = 0.2, position = position_jitter(0.2)) +
   stat_pvalue_manual(pwc, hide.ns = TRUE, label = "p.adj.signif", tip.length = 0) +

   xlab(NULL) + ylab("Thalamic strength") +
   theme_classic() +
   theme(plot.title = element_text(size = 12, face = "bold", hjust = 0.5)) +
   theme(axis.title = element_text(size = 9, face = "bold"),
         axis.text.x = element_text(colour = "black", size = 9, face = "bold"),
         axis.text.y = element_text(colour = "black", size = 7)) +
   theme(legend.text = element_text(size = 12),
         legend.title=element_blank(),
         legend.margin = margin(1,1,1,1, unit = "mm"),
         legend.direction = "horizontal",
         legend.position = "none") +
   scale_y_continuous(breaks = seq(0, 0.1, 0.01), limits = c(0.01, 0.04)) +
   scale_fill_paletteer_d("rcartocolor::Pastel"))
png("boxplot_gamma_sleep_R2Mean_engel.png", width = 6, height = 4, units = "cm",
   res = 700)
print(p)
dev.off()

# Figure 3c FC beta band vs engel score - Sleep #
data_beta$engel_class_code <- as.numeric(data_beta$engel_class_code)
spearman_CI(data_beta$FC_sleep_beta, data_beta$engel_class_code)
(p <- ggscatter(data = data_beta,
               x = "engel_class_code", y = "FC_sleep_beta",
               size = 0, add = "reg.line", conf.int = T,
               add.params = list(color = "blue", fill = "lightgray",
                                 size = 0.5)) +
  geom_point(size = 0.3) +
  stat_cor(method = "spearman", label.x = 2.6, label.y = 0.08, size = 2)  +
  xlab("Engel score") + ylab("Thalamic strength") +
  theme_classic() +
  theme(plot.title = element_text(size = 12, face = "bold", hjust = 0.5)) +
```

```
  theme(axis.title = element_text(size = 9, face = "bold"),
        axis.text = element_text(colour = "black", size = 7)) +
  theme(legend.text = element_text(size = 12),
        legend.title=element_blank(),
        legend.margin = margin(1,1,1,1, unit = "mm"),
        legend.direction = "horizontal",
        legend.position = "bottom") +
  scale_x_continuous(breaks = seq(1, 4, 1), limits = c(1, 4)) +
  scale_y_continuous(breaks = seq(0, 0.08, 0.01), limits = c(0.02, 0.08)))
png("corr_beta_sleep_R2Mean_engel.png", width = 6, height = 4, units = "cm",
    res = 700)
print(p)
dev.off()

# Figure 3d FC delta band vs duration - Rest #
data_delta$duration <- as.numeric(data_delta$duration)
spearman_CI(data_delta$FC_rest_delta, data_delta$duration)
(p <- ggscatter(data = data_delta,
                x = "duration", y = "FC_rest_delta",
                size = 0, add = "reg.line", conf.int = T,
                add.params = list(color = "blue", fill = "lightgray", size = 0.5)) +
  geom_point(size = 0.3) +
  stat_cor(method = "spearman", label.x = 32, label.y = 0.22, size = 2)  +
  xlab("Duration (years)") + ylab("Thalamic strength") +
  theme_classic() +
  theme(plot.title = element_text(size = 12, face = "bold", hjust = 0.5)) +
  theme(axis.title = element_text(size = 9, face = "bold"),
        axis.text = element_text(colour = "black", size = 7)) +
  theme(legend.text = element_text(size = 12),
        legend.title=element_blank(),
        legend.margin = margin(1,1,1,1, unit = "mm"),
        legend.direction = "horizontal",
        legend.position = "bottom") +
  scale_x_continuous(breaks = seq(0, 55, 5), limits = c(0, 55))  +
  scale_y_continuous(breaks = seq(0, 0.22, 0.02), limits = c(0.08, 0.22)))
png("corr_delta_rest_R2Mean_duration.png", width = 6, height = 4, units = "cm",
    res = 700)
print(p)
```

```
# Figure 3e FC broadband vs duration - Rest #
data_broadband$duration <- as.numeric(data_broadband$duration)
spearman_CI(data_broadband$FC_rest_broadband, data_broadband$duration)
(p <- ggscatter(data = data_broadband,
            x = "duration", y = "FC_rest_broadband",
            size = 0, add = "reg.line", conf.int = T,
            add.params = list(color = "blue", fill = "lightgray", size = 0.5)) +
  geom_point(size = 0.3) +
  stat_cor(method = "spearman", label.x = 32, label.y = 0.14, size = 2)  +
  xlab("Duration (years)") + ylab("Thalamic strength") +
  theme_classic() +
  theme(plot.title = element_text(size = 12, face = "bold", hjust = 0.5)) +
  theme(axis.title = element_text(size = 9, face = "bold"),
      axis.text = element_text(colour = "black", size = 7)) +
  theme(legend.text = element_text(size = 12),
      legend.title=element_blank(),
      legend.margin = margin(1,1,1,1, unit = "mm"),
      legend.direction = "horizontal",
      legend.position = "bottom") +
  scale_x_continuous(breaks = seq(0, 55, 5), limits = c(0, 55))  +
  scale_y_continuous(breaks = seq(0, 0.22, 0.02), limits = c(0.0, 0.14)))
png("corr_broadband_rest_R2Mean_duration.png", width = 6, height = 4, units = "cm",
  res = 700)
print(p)

# Figure 3f FC broadband vs age at SEEG - Rest #
data_broadband$age_at_SEEG <- as.numeric(data_broadband$age_at_SEEG)
spearman_CI(data_broadband$FC_rest_broadband, data_broadband$age_at_SEEG)
(p <- ggscatter(data = data_broadband,
            x = "age_at_SEEG", y = "FC_rest_broadband",
            size = 0, add = "reg.line", conf.int = T,
            add.params = list(color = "blue", fill = "lightgray", size = 0.5)) +
 geom_point(size = 0.3) +
 stat_cor(method = "spearman", label.x = 42, label.y = 0.14, size = 2)  + # Add correlation coefficient
 xlab("Age at SEEG (years)") + ylab("Thalamic strength") +
 theme_classic() +
 theme(plot.title = element_text(size = 12, face = "bold", hjust = 0.5)) +
 theme(axis.title = element_text(size = 9, face = "bold"),
     axis.text = element_text(colour = "black", size = 7)) +
```

```r
  theme(legend.text = element_text(size = 12),
        legend.title=element_blank(),
        legend.margin = margin(1,1,1,1, unit = "mm"),
        legend.direction = "horizontal",
        legend.position = "bottom") +
  scale_x_continuous(breaks = seq(0, 70, 10), limits = c(0, 70))  +
  scale_y_continuous(breaks = seq(0, 0.14, 0.02), limits = c(0, 0.14)))
png("corr_broadband_rest_R2Mean_age_at_SEEG.png", width = 6, height = 4,
    units = "cm", res = 700)
print(p)
dev.off()

##########################################################################################################################
######################## Additional Analyses ##########################################



####Grouping per Engel 1-2 vs 3-4####
#Subset for Engel 1-2
group1year_Engel12 <- data[data$Engel_1_year <2.2,]


#Subset for Engel 3-4
group1year_Engel34 <- data[data$Engel_1_year >2.3,]


data$group <- ifelse(data$Engel_1_year >= 2.3, "group1_year_Engel34", "group1_year_Engel12")


# Perform the Wilcoxon test tra gruppi di outcome Engel
wilcox_test <- wilcox.test(data$sleep_rate_sleep ~ group, data = data)
print(wilcox_test)


####Differenza Engel 12 mesi e Engel ultimo FU####
# Perform Wilcoxon signed-rank test
result <- wilcox.test(data$Engel_1_year, data$Engel_last_FU, paired = TRUE)
print(result)


####Grouping per Engel 1 vs 2-4####
#Subset for Engel 1
group1year_Engel1 <- data[data$Engel_1_year <1.2,]


#Subset for Engel 2-4
```

```
group1year_Engel24 <- data[data$Engel_1_year >1.3,]

data$group2 <- ifelse(data$Engel_1_year >= 1.3, "group1year_Engel24", "group1year_Engel1")

# Perform the Wilcoxon test tra gruppi di outcome Engel
wilcox_test <- wilcox.test(data$gamma_r2_sleep ~ group2, data = data)

# Print the results
print(wilcox_test)



##############################################################################
#####################################################################################
#############################################################################
        ##    ONLY THERMO   ##
data <- Engel_12_months_added_only_thermo

####TUTTE LE CORRELAZIONI####

##Fig 2##

# Test di correlazione di Spearman con p-value
spearman_test <- cor.test(data$Engel_last_FU, data$beta_r2_sleep, method = "spearman")
print(spearman_test)

# Grafico con base R
plot(data$Engel_12_months, data$beta_r2_sleep, pch = 19, col = "blue")
abline(lm(data$beta_r2_sleep ~ data$Engel_12_months), col = "red", lwd = 2)

####Shapiro su Engel_ultimo_FU e su Engel_12_mesi####
shapiro.test(data$Engel_last_FU)
shapiro.test(data$Engel_12_months)



####Grouping per Engel 1-2 vs 3-4####
#Subset for Engel 1-2
grouplastFU_Engel12 <- data[data$Engel_last_FU <2.2,]

#Subset for Engel 3-4
```

```
grouplastFU_Engel34 <- data[data$Engel_last_FU >2.3,]

data$group <- ifelse(data$Engel_last_FU >= 2.3, "grouplastFU_Engel34", "grouplastFU_Engel12")

# Perform the Wilcoxon test tra gruppi di outcome Engel
wilcox_test <- wilcox.test(data$HFO_rate_sleep ~ group, data = data)

# Print the results
print(wilcox_test)

####Differenza Engel 12 mesi e Engel ultimo FU####
# Perform Wilcoxon signed-rank test
result <- wilcox.test(data$Engel_12_months, data$Engel_last_FU, paired = TRUE)

# Display the result
print(result)

####Grouping per Engel 1 vs 2-4####
#Subset for Engel 1
grouplastFU_Engel1 <- data[data$Engel_last_FU <1.2,]

#Subset for Engel 2-4
grouplastFU_Engel24 <- data[data$Engel_last_FU >1.3,]

data$group2 <- ifelse(data$Engel_last_FU >= 1.3, "grouplastFU_Engel24", "grouplastFU_Engel1")

# Perform the Wilcoxon test tra gruppi di outcome Engel
wilcox_test <- wilcox.test(data$sleep_rate_sleep ~ group2, data = data)

# Print the results
print(wilcox_test)
```

Sub	position	code_epilepsy_type	code_aetiology	lateralisation	sex	age_at_SEEG	epilepsy_onset	duration	surgery_code	thermo	follow_up_months	engel_class_code	max_spike_rate_thalamus_rest	max_spike_rate_thalamus_sleep	max_HFO_rate_thalamus_rest	max_HFO_rate_thalamus_sleep
1	pulv	biT	crypto	B	F	22	4	18	contro	yes	21	3	0	0.4	0	4.7
2	pulv	Tplus	MCD	L	F	51	5	46	contro	yes	36	4	0.8	0.2	0	0
3	thal	PE	MCD	R	F	35	12	23	yes	yes	22	4	0.3	0.2	0.1	0
4	thal	TLE;MTLE	NDT	R	F	26	23	3	yes	no	18	1	0.2	0.8	0	0.7
5	pulv	TLE;MTLE	crypto	L	F	37	25	12	yes	yes	42	1	0.1	0.3	1.2	1.6
6	pulv;ant	IE	crypto	R	M	42	12	30	contro	yes	19	2	1.9	0	0	0
7	thal	PE	poststroke	R	M	10	0	10	contro	yes	18	3	0.3	1.1	0.1	1.7
8	thal	TLE;MTLE	crypto	R	M	60	36	24	yes	no	69	2	0	0.4	0.1	0.1
9	pulv	Tplus	MCD	R	F	38	20	18	yes	yes	20	1	0.9	2.2	0	0
10	pulv	biOp	Inf	B	F	38	3	35	contro	yes	8	3	0.1	0.1	0.1	0.8
11	pulv	biT	crypto	B	F	27	10	17	contro	yes	33	1	0.4	0.1	0	0
12	pulv	Tplus	MCD	L	M	8	0.58	4.42	no	yes	7	3	1.9	7.3	0.8	0
13	pulv	TLE;MTLE	MCD	R	F	42	33	9	no	yes	42	1	0	0	0	0.1
14	pulv;ant	TLE;MTLE	HS	L	M	35	20	15	yes	yes	19	1	6.5	10.9	0	3.8
15	Rthal;Lpulv	biT	crypto	B	F	37	21	16	contro	yes	6	2	0	0.3	0	0.2
16	pulv	IOpE	MCD	R	F	20	14	6	no	yes	40	1	0	0.2	0	0
17	thal	TLE;LTLE	crypto	R	M	21	15	6	yes	no	10	2	0	0.2	0	0.3
18	thal	TLE;MLTLE	HS	R	M	7	4	3	yes	yes	12	1	2.4	0.3	0	0
19	pulv	TLE;MTLE	cavernoma	L	F	33	22	11	yes	yes	no FU	no FU	1.8	5.1	0	0
20	pulv	Tplus	MCD	L	M	6	1.25	4.75	no	yes	no FU	no FU	0	0.5	0	0
21	pulv	IOpE	crypto	L	F	36	5	31	no	yes	2	1	0.2	0.6	0.4	0
22	thal	Tplus	crypto	L	M	19	10	9	contro	no	120	4	0.1	0.5	0.2	2
23	pulv	TLE;LTLE	HS	L	F	37	10	24	yes	yes	23	1	0	3.2	0	0
24	thal ant lat	Mot	HH	R	M	10	5	5	contro	yes	19	3	3	5.5	0	0
25	pulv	biT	MCD	B	F	26	15	11	contro	yes	16	3	0.4	2.1	0	0
26	pulv	Mot	crypto	R	M	35	1	34	contro	yes	76	3	0.7	1	6.4	0.3
27	pulv	Mot	MCD	L	M	16	0.5	15.5	no	yes	12	1	0.9	1.4	0	1.7
28	pulv	TLE;MLTLE	crypto	L	M	24	12	12	yes	yes	24	2	1.3	1.4	0	0
29	thal	OE	poststroke	R	M	18	13	5	no	yes	42	2	0.9	0.1	0	0
30	pulv	biT	crypto	B	F	47	5	42	contro	yes	no FU	no FU	9.9	3.8	0	0
31	pulv	Tplus	crypto	R	F	39	20	19	contro	no	100	3	0.3	9	0	0.7
32	pulv	biT	crypto	B	F	23	5	18	contro	no	106	2	8.2	6.2	0	0.2
33	pulv	Tplus	crypto	L	M	57	42	15	contro	no	41	3	7	4.6	0	0
34	pulv	biT	crypto	B	F	30	18	12	contro	yes	2	3	0.1	0.1	0	0
35	thal	TLE;MTLE	crypto	L	M	45	17	28	contro	yes	11	1	0	0.4	0	0.1
36	pulv	IE	MCD	R	M	29	3	26	yes	yes	4	1	0.2	0.1	0	0
37	pulv	Tplus	cavernoma	R	M	36	23	13	no	yes	23	1	0.1	0.4	0.1	0
38	thal	TLE;BTLE	MCD	L	F	11	3	8	yes	yes	5	1	2.6	0.8	0	0
39	thal	TLE;LTLE	MCD	L	M	3	2	1	no	yes	14	1	3.9	1.6	2.7	0
40	pulv	TLE;MTLE	crypto	L	M	52	35	17	no	no	no FU	no FU	0.2	1.1	0.4	5.8
41	thal	OpE	poststroke	L	F	17	11	6	contro	yes	18	3	1.3	2.8	0	0
42	thal	OpE	crypto	R	M	13	9	4	no	yes	20	2	0.4	0.2	1.2	0
43	pulv	Tplus	HS	R	M	53	47	6	yes	no	5	1	0.4	2.5	0	0
44	pulv	Mot	poststroke	L	F	7	2	5	yes	yes	54	2	1.9	0.2	0.2	0.9
45	pulv	IOpE	crypto	L	M	23	13	10	yes	yes	no FU	no FU	0	0.2	0	0
46	Rpulv;Lthal	TLE;MLTLE	crypto	L	M	28	11	17	contro	yes	8	4	0.9	0.4	0	0
47	pulv	TLE;LTLE	crypto	L	F	46	25	21	no	yes	2	1	0.1	1.6	0	0
48	pulv	TLE;MTLE	poststroke	R	F	49	40	9	yes	no	26	1	3.4	2.6	0	0
49	thal	PE	Inf	L	M	14	1.42	12.58	no	yes	12	3	0	1.1	0	0
50	thal	TLE;BTLE	Inf	L	M	51	10	41	no	no	no FU	no FU	0	0	0	0
51	thal	OpE	MCD	R	F	20	0	20	yes	yes	no FU	no FU	0.2	0	0	0
52	pulv	Tplus	MCD	R	M	9	0.33	8.66	yes	no	12	1	0.3	0.2	0	0.1
53	pulv	PE	MCD	L	M	21	0.33	20.67	contro	yes	7	4	0.7	0.4	0	0.7
54	thal	TLE;MTLE	HS	R	F	32	24	8	yes	yes	33	1	1.2	0.2	0.1	0.4
55	pulv	IE	NDT	R	F	21	14	7	yes	no	40	3	0.2	0.6	0	0
56	thal	Mot	MCD	L	F	9	0.25	8.75	contro	yes	42	4	0.3	0.2	0	2
57	thal;ant	Tplus	TC	R	F	56	35	21	contro	yes	35	3	2.8	0.7	12.7	0
58	thal	Mot	crypto	R	F	17	15	2	contro	yes	7	2	0	0.9	0	0.1
59	pulv	TLE;MTLE	HS	L	F	70	20	51	yes	yes	40	1	1.4	1.4	0	0
60	pulv	TLE;MTLE	MCD	L	M	7	1.5	5.5	yes	no	13	2	1.3	0.1	0	0
61	pulv	Tplus	Inf	R	F	37	20	17	contro	yes	3	2	0	0.3	0	0
62	pulv	TLE;MTLE	HS	L	M	26	5	21	yes	yes	10	1	0	5.6	0	2
63	pulv	Tplus	TC;NDT	R	M	23	11	12	yes	yes	5	1	0	0.3	0	0
64	pulv	biT	MCD	B	F	30	21	9	contro	no	9	1	7	10.1	0	0
65	pulv	TLE;MTLE	MCD	L	M	28	10	18	contro	no	105	2	0	0.4	0.1	0.1
66	thal	Tplus	TC	L	M	13	6	7	contro	yes	17	2	0.6	0.6	0	0
67	pulv	Tplus	crypto	L	F	54	23	31	contro	no	48	3	0.1	4.8	0	0
68	pulv	PE	crypto	R	M	16	10	6	contro	yes	14	1	14.6	0.6	0	0
69	thal	biT	crypto	B	M	33	11	22	contro	yes	22	2	0	0.5	0	0.1
70	thal	Tplus	MCD	R	M	3	1.08	1.92	yes	yes	21	1	0.8	0	0	0
71	Rthal;Lpulv	biT	crypto	B	M	5	0.5	4.5	yes	no	11	1	0.1	0.9	0	0.2
72	Rpulv;Lthal	TLE;MTLE	crypto	R	M	37	22	15	no	yes	6	1	1.4	0.3	0	0
73	pulv	Tplus	crypto	L	M	21	9	12	contro	yes	30	2	0.4	0.1	0.1	0.3
74	pulv	TLE;MTLE	TC	L	F	43	28	15	yes	yes	7	2	1.5	0.6	0	0
75	pulv	TLE;BTLE	DEPDC5	R	M	9	4	5	no	yes	17	3	1	1.4	0	1.7
76	pulv	IOpE	MCD	L	F	25	21	4	contro	yes	7	3	0	0.4	0	
77	thal	Tplus	TC	R	M	47	22	25	yes	yes	17	1	0.4	1.5	0	0.5
78	pulv	IE	MCD	L	M	7	2	5	no	yes	18	3	26.6	5	0	4.9
79	pulv	TLE;MTLE	HH	L	M	23	0.5	22.5	no	yes	no FU	no FU	0	2.8	0	0
80	pulv		crypto	R	M	32	6	26	no	no	21	1	0.1	0.4	0	0
81	thal;ant	IE	HH	R	F	14	7	7	no	yes	7	1	0.6	1	0	0
82	pulv	Tplus	crypto	R	F	10	5	5	yes	yes	9	1	0.5	1.5	0	0
83	pulv	TLE;MTLE	HS	R	F	20	8	12	no	no	66	4	0	1.4	0	0.7
84	pulv	TLE;LTLE	crypto	L	F	25	11	14	yes	yes	30	3	0.1	0.1	0.5	0
85	pulv	TLE;MTLE	NDT	L	F	48	11	37	no	yes	14	1	1.7	0.4	0.9	0.4
86	pulv	Tplus	crypto	R	F	38	19	19	contro	yes	72	3	0.5	2.5	0	0.7
87	pulv	Mot	NDT	L	F	32	10	10	yes	yes	27	2	1.9	1.7	0	0
88	pulv	TLE;MLTLE	crypto	L	M	29	7	22	yes	yes	66	3	2.2	3.8	0.4	0.5
89	pulv	Tplus	NDT	L	M	22	12	10	no	yes	20	2	0	0.2	0	0
90	thal	OE	MCD	R	M	15	10	5	contro	yes	2	2	0.5	0.1	0	0
91	pulv	Mot	TC	R	M	24	17	7	contro	no	no FU	no FU	1	0.2	0	0
92	pulv	TLE;MTLE	crypto	L	F	47	38	9	yes	yes	6	1	0	0.2	0	0.2
93	pulv	TLE;MLTLE	TC	R	F	42	24	18	no	yes	24	3	2.1	0.8	0.8	1.4
94	pulv	TLE;MTLE	crypto	L	M	30	3	27	yes	yes	28	2	0	0.1	0	0
95	pulv	Tplus	NDT	L	F	48	8	40	yes	yes	25	1	0	0.2	0	1
96	thal	TLE;BTLE	MCD	R	F	29	8	21	yes	no	17	1	0.1	0.2	0.1	1.2
97	pulv	biT	MCD	B	F	29	0.75	28.25	contro	yes	11	2	0	0.1	0	0
98	pulv	Tplus	crypto	R	F	54	36	18	contro	yes	10	4	1.1	2	0	0
99	pulv	TLE;LTLE	crypto	R	F	22	13	9	yes	yes	8	1	0.2	2	0	0.2
100	thal	biT	MCD	B	F	29	20	9	contro	yes	5	4	0.1	1.5	0	0
101	thal	biT	MCD	B	F	17	13	4	contro	yes	12	2	0	0.1	0.1	0
102	thal	Tplus	HS	R	F	45	0.66	44.34	contro	yes	84	2	0	0.4	0	4.1
103	pulv	TLE;MTLE	crypto	L	M	29	3	26	no	yes	35	3	2.3	5.3	0	0
104	pulv	TLE;LTLE	MCD	L	F	23	15	8	contro	yes	20	4	10.3	2	0.8	0.4
105	pulv	Tplus	crypto	R	F	18	13	5	yes	yes	30	2	1.1	0.5	0	0
106	pulv	OpE	crypto	R	M	49	14	35	yes	yes	6	1	0	0	0	0
107	thal	TLE;MTLE	crypto	L	M	15	11	4	yes	yes	24	1	15.6	3.4	0	0
108	pulv	TLE;MTLE	crypto	R	F	42	24	18	yes	yes	15	2	1.1	0.5	0	1.5
109	pulv	Tplus	MCD	R	M	7	3	4	no	yes	12	1	3.6	5.8	0	0
110	pulv	Tplus	Inf	B	M	29	0.58	28.42	contro	no	55	2	1.5	0.3	0	0.1
111	pulv	PE	TC	B	M	10	9	1	contro	yes	18	2	10.8	6.3	0	0
112	pulv	TLE;MTLE	crypto	R	M	31	22	9	yes	yes	4	1	0.3	0.5	0	0
113	pulv	TLE;MTLE	crypto	R	M	39	31	8	yes	yes	6	1	3.4	6.4	0	0
114	pulv;ant	biT	crypto	B	F	52	30	22	contro	yes	32	3	3	6.8	0	0
115	pulv	PE	MCD	L	M	22	21	1	no	yes	39	2	0.4	0.3	0	0.1
116	pulv	TLE;MTLE	crypto	R	M	35	28	7	yes	yes	3	1	1.4	0.8	0	0
117	pulv	TLE;MTLE	crypto	R	F	41	34	7	yes	no	1.5	1	0.7	2.4	0	0
118	pulv	Mot	crypto	L	M	15	3	12	contro	yes	31	4	1.1	0.8	0.1	0.3
119	pulv	TLE;MTLE	HS	L	M	10	3	7	yes	yes	18	1	0	0	0	0
120	thal	Mot	NDT	L	F	30	12	18	yes	yes	36	1	2.8	2.8	0	0
121	pulv	IE	MCD	R	M	31	9	22	yes	yes	6	2	0.2	3.2	0	0

Sub	max_spike_rate_normalized_thalamus_rest	max_HFO_rate_normalized_thalamus_rest	max_spike_rate_normalized_thalamus_sleep	max_HFO_rate_normalized_thalamus_sleep	thVSnonPZ_beta_r2_sleep_mean	thVSnonPZ_broadband_r2_rest_mean	thVSnonPZ_delta_r2_rest_mean	thVSnonPZ_gamma_r2_sleep_mean
1	0		0.023121387	0.734375	0.054272214	0.042209285	0.102226691	0.014682346
2	0.069565217		0.006666667		0.048286609	0.035324351	0.101089521	0.016332955
3	0.005905512	0.002320186	0.004608295	0	0.03959932	0.031304324	0.098526111	0.01633829
4	0.036363636	0	0.026755853	0.025454545	0.042998476	0.043162608	0.113665649	0.016814994
5	0.005	0.011019284	0.012658228	0.64	0.049964527	0.03005105	0.102170782	0.015350515
6	0.240506329		0		0.036844878	0.062150794	0.122127337	0.014645787
7	0.009404389	0.011627907	0.042145594	0.024285714	0.045542541	0.057746439	0.114912356	0.016090297
8	0	0.00154321	0.020618557	0.002398082	0.03503001	0.053368588	0.094560271	0.01432257
9	0.062937063		0.075085324		0.048806777	0.084379997	0.139865511	0.020288909
10	0.090909091	0.010309278	0.052631579	0.041666667	0.058988341	0.035103951	0.108850097	0.014865569
11	0.007518797	0	0.002702703		0.037076785	0.041404458	0.107363306	0.01476579
12	0.025641026	0.008255934	0.13594041		0.04768544	0.119193366	0.190242532	0.026077066
13	0	0	0	0.058823529	0.034930201	0.043384102	0.107962469	0.014132266
14	0.321782178		0.313218391	0.075396825	0.040132484	0.067787591	0.124602846	0.018329616
15	0	0	0.012875536	0.003984064	0.035505301	0.028997035	0.10790557	0.016303424
16	0		0.008064516		0.054276211	0.035734159	0.107477583	0.014436795
17	0	0	0.035714286	0.020833333	0.041753442	0.040789263	0.100494983	0.013940509
18	0.188976378		0.015306122		0.032670935	0.047382109	0.109805118	0.013819306
19	0.066420664		0.115909091		0.039125051	0.040059218	0.102474635	0.016332688
20	0		0.051546392		0.037145342	0.047906042	0.107931957	0.015701911
21	0.285714286	0.008438819	0.125		0.064546519	0.06345462	0.140786175	0.015923411
22	0.02	0.014084507	0.080645161	0.540540541	0.052110099	0.044350281	0.105275927	0.013905686
23	0		0.542372881		0.035077119	0.040654422	0.120378544	0.013902341
24	0.222222222		0.317919075		0.043951651	0.086450017	0.123178351	0.020882279
25	0.016194332		0.067307692		0.060958518	0.061894942	0.122791018	0.017584547
26	0.026819923	0.115315315	0.076923077	0.009174312	0.049698475	0.053530565	0.109503973	0.017434499
27	0.016759777		0.023728814	0.17	0.045403284	0.041540878	0.104553074	0.016576052
28	0.464285714		0.074468085		0.041781935	0.05689762	0.104392801	0.016068367
29	0.1		0.005494505		0.034600192	0.040053097	0.094341908	0.014635221
30	0.269754768		0.120253165		0.042229378	0.052826561	0.106613451	0.017106925
31	0.028037383	0	0.179640719	0.0155902	0.058334776	0.063133728	0.136086859	0.0243061
32	0.165656566	0	0.206666667	0.039215686	0.056758351	0.07344628	0.13104316	0.03341004
33	0.184210526		0.171003717		0.043700088	0.07305949	0.13809572	0.01853808
34	0.022222222		0.006622517		0.052896925	0.040728033	0.10490477	0.014740238
35	0	0	0.016806723	0.002283105	0.037005339	0.057716082	0.110690588	0.012805188
36	0.002280502		0.001023541		0.032602408	0.053916016	0.114790912	0.013977091
37	0.0078125	0.012195122	0.011730205	0	0.039805728	0.037152752	0.09414403	0.016370631
38	0.078313253		0.04519774		0.032979374	0.066438014	0.129627181	0.014083634
39	0.102362205	0.054	0.075829384	0	0.037971689	0.122153698	0.168335461	0.016020437
40	0.02739726	0.008213552	0.044534413	0.3625	0.039792839	0.055454846	0.111025718	0.017329153
41	0.097744361		0.304347826		0.062167902	0.053743167	0.123301289	0.017471597
42	0.285714286	0.155844156	0.016260163		0.065971173	0.041450136	0.1136535	0.014447367
43	0.01793722		0.039494471		0.05059243	0.074088555	0.152637249	0.017941747
44	0.1484375	0.002496879	0.046511628	0.013867488	0.03879464	0.049354709	0.112791958	0.013755538
45	0		0.010869565		0.037667061	0.052116183	0.113963441	0.014390107
46	0.027272727		0.020833333		0.041299624	0.040340376	0.102113279	0.014541393
47	0.025641026		0.095808383		0.05034729	0.050187502	0.12701712	0.015991582
48	0.219354839		0.087248322		0.04578071	0.052548948	0.12161703	0.021526173
49	0	0	0.055555556		0.054253588	0.079653986	0.097294065	0.015626663
50	0		0		0.04012098	0.047942757	0.100680886	0.014171372
51	0.022727273		0		0.043790129	0.036656155	0.104388596	0.014133716
52	0.005545287		0.003703704	0.001538462	0.037027157	0.067000113	0.124334863	0.014049981
53	0.024390244	0	0.027210884	0.118644068	0.048180347	0.043365419	0.10879334	0.015914104
54	0.080536913	0.001886792	0.010362694	0.017021277		0.041573676	0.105398275	0.013919959
55	0.037037037		0.333333333		0.071684601	0.06690538	0.123453403	0.018826583
56	0.013333333		0.013157895	0.243902439	0.037403578	0.055097723	0.109912452	0.014514626
57	0.088050314	0.081358104	0.033018868		0.052208699	0.043115221	0.138017059	0.018406876
58	0	0	0.333333333	0.125	0.038163715	0.034000576	0.105258224	0.014306096
59	0.259259259		0.093959732		0.031794505	0.042502581	0.101779331	0.013357252
60	0.317073171		0.013888889		0.042851606	0.052636282	0.116363197	0.013223853
61	0		0.008928571		0.060348243	0.037433139	0.095178763	0.014296182
62	0	0	0.225806452	0.089686099	0.045973365	0.040158661	0.102172461	0.018819352
63	0		0.009803922		0.044005282	0.039635282	0.103236784	0.015450606
64	0.486111111		0.417355372		0.037407338	0.039805456	0.112066493	0.016732698
65	0	0.01	0.5	0.333333333	0.040686718	0.052918353	0.105941159	0.015683793
66	0.095238095		0.050420168		0.033082811	0.055172257	0.110611132	0.014153002
67	0.009259259		0.444444444		0.064511907	0.031379117	0.085827028	0.018469037
68	1		0.020979021		0.042339406	0.049942103	0.099959049	0.014002309
69	0	0	0.057471264	0.015384615	0.041901717	0.037380563	0.105860796	0.014418307
70	0.285714286		0		0.028507983	0.051400325	0.100871978	0.014884842
71	0.015151515	0	0.214285714	0.007968127	0.031825567	0.048275723	0.100031569	0.014582842
72	0.14893617		0.046153846		0.031942228	0.041444196	0.105154011	0.013807069
73	0.142857143	0.007092199	0.018181818	0.031578947	0.045218611	0.04704123	0.094793661	0.01443468
74	0.104166667	0	0.053571429	0	0.036929214	0.042054264	0.10496294	0.013861029
75	0.039525692		0.067961165	0.116438356	0.04422212	0.063120169	0.130266016	0.014166271
76	0		0.074074074		0.05880158	0.077684244	0.101568513	0.019854153
77	0.097560976	0	0.104166667	0.01002004	0.043471992	0.052527941	0.111276432	0.018472562
78	0.204144282		0.162337662	0.215859031	0.055772428	0.067829831	0.140001494	0.020244402
79	0	0	0.4375		0.062706079	0.034088122	0.106289086	0.017109682
80	0.024390244		0.021164021		0.050563315	0.037174016	0.101983248	0.015618256
81	0.272727273		0.333333333		0.044229865	0.092665152	0.146147368	0.014305627
82	0.037593985		0.048231511		0.038559695	0.042947536	0.10177087	0.016664052
83	0	0	0.0875	0.095890411	0.044311298	0.034655811	0.106810836	0.015284353
84	0.011111111	0.012787724	0.004115226	0	0.035823028	0.039107387	0.097689298	0.015453048
85	0.033530572	0.012765957	0.011204482	0.021164021	0.035267954	0.044765893	0.104476701	0.014001305
86	0.011961722	0	0.373134328	0.056910569	0.043698902	0.052156109	0.114526167	0.016443426
87	0.115151515		0.082524272		0.039893185	0.112020632	0.155445943	0.018170766
88	0.144736842	0.007393715	0.111111111	0.06097561	0.044330413	0.044287852	0.098022658	0.015051122
89	0		0.022988506		0.039623827	0.039568418	0.099046006	0.013978206
90	0.019157088		0.002739726		0.03349719	0.053335083	0.114132591	0.012893344
91	0.243902439		0.015384615		0.030204937	0.055423535	0.112610326	0.013021711
92	0	0	0.040816327	0.076923077	0.042417619	0.048357616	0.105611594	0.01484626
93	0.031019202	0.012965964	0.02507837	0.04620462	0.043103384	0.046817034	0.110227698	0.016005258
94	0		0.010989011		0.038637148	0.040253444	0.100702349	0.013683351
95	0	0	0.004796163	0.03875969	0.044244697	0.037234445	0.098028343	0.017799006
96	0.00055991	0.00255102	0.001309758	0.089552239	0.032297689	0.047801605	0.099215638	0.014078106
97	0	0	0.007142857		0.06173746	0.036503722	0.101660144	0.014665844
98	0.06626506		0.085106383		0.035241386	0.053672132	0.111938714	0.017767453
99	0.030769231	0	0.083333333	0.015384615	0.038448507	0.040890365	0.112077937	0.016836823
100	0.007692308	0	0.136363636	0	0.038226877	0.040016459	0.107694068	0.013766523
101	0	0.038461538	0.006756757		0.034635111	0.029031302	0.108985217	0.014485451
102	0	0	0.009661836	0.539473684	-2.63158E+97		0.098859347	-2.63158E+97
103	0.142857143	0	0.201520913		0.041042361	0.04545437	0.110317796	0.014422031
104	0.578651685	0.083333333	0.21978022	0.012461059	0.045428796	0.089754248	0.158558543	0.017521425
105	0.089430894		0.032258065		0.029244255	0.038781997	0.098717674	0.017514261
106	0		0		0.04209807	0.028938299	0.097558804	0.01489693
107	0.968944099		0.918918919		0.032321474	0.042768559	0.105652406	0.013790893
108	0.125	0	0.024154589	0.154639175	0.032027083	0.040908659	0.106466669	0.014854855
109	0.153846154		0.120833333		0.063901761	0.108705264	0.172763482	0.024425798
110	0.148514851	0	0.014925373	0.083333333	0.040358225	0.047512762	0.111755078	0.017234998
111	0.249422633		0.11130742		0.055999524	0.070000331	0.110570771	0.020342536
112	0.020134228		0.04587156		0.042028578	0.058599847	0.10604937	0.014152074
113	0.047954866		0.095952024		0.043948683	0.05506897	0.116530589	0.019070128
114	0.198675497		0.369565217		0.041635683	0.040836976	0.105976438	0.018326782
115	0.018099548		0.09375	0.166666667	0.038300727	0.039933986	0.103560058	0.014786418
116	0.538461538		0.033195021		0.040660031	0.046113653	0.128083267	0.015932576
117	0.020408163		0.042031524		0.041451417	0.051707502	0.118914942	0.018061411
118	0.026190476	0.004608295	0.023054755	0.021582734	0.046466347	0.060631933	0.112992388	0.022297443
119	0	0	0	0	0.039227569	0.048730161	0.12639386	0.015370997
120	0.056451613		0.082840237		0.053540838	0.051140117	0.104568848	0.022124858
121	0.003831418		0.112676056		0.03922113	0.041234117	0.096268188	0.01616618

Sub	thVSnonth_beta_r2_sleep_mean	thVSnonth_broadband_r2_rest_mean	thVSnonth_delta_r2_rest_mean	thVSnonth_gamma_r2_sleep_mean	thVSPZ_beta_r2_sleep_mean	thVSPZ_broadband_r2_rest_mean	thVSPZ_delta_r2_rest_mean	thVSPZ_gamma_r2_sleep_mean
1	0.052506612	0.042839323	0.102437207	0.014629074	0.043510449	0.046188472	0.103556261	0.014357639
2	0.04721455	0.035534723	0.101052783	0.016205093	0.040567782	0.036837021	0.100825357	0.015412348
3	0.038971308	0.031352186	0.099016479	0.01619459	0.032586518	0.031782942	0.103429786	0.014733646
4	0.046763083	0.043875207	0.115259089	0.017961598	0.057900045	0.046161462	0.120371378	0.021353634
5	0.049964527	0.03005105	0.102170782	0.015350515				
6	0.036832977	0.062017517	0.12197851	0.014635472	0.036661599	0.060200103	0.119949048	0.014486932
7	0.044171965	0.057361362	0.113819683	0.015972833	0.039179153	0.055667021	0.109011922	0.015544927
8	0.035246474	0.059232595	0.099419173	0.014587156	0.035592818	0.067577528	0.106333764	0.015010494
9	0.048617489	0.084993926	0.139829614	0.020230942	0.041803131	0.107504664	0.138513375	0.018144147
10	0.059239104	0.034946982	0.112653424	0.015034124	0.059364485	0.034875632	0.114382209	0.015118401
11	0.037424603	0.04350758	0.109190907	0.014937895	0.039053857	0.048648543	0.113658375	0.015744067
12	0.048883002	0.123058226	0.196777259	0.027341916	0.050789324	0.129152811	0.207082019	0.029355352
13	0.034878492	0.043002942	0.107405509	0.014173875	0.034309696	0.039343807	0.102058691	0.01463157
14	0.040945502	0.067990678	0.125289514	0.019378234	0.049617692	0.071443158	0.136962878	0.03056349
15	0.035903896	0.029185491	0.106820813	0.016253204	0.03624021	0.029786194	0.103363152	0.016210831
16	0.049934402	0.034866954	0.104794311	0.01435988	0.043619044	0.0335455	0.100705514	0.014248005
17	0.041339529	0.040701357	0.100869453	0.013944065	0.034854879	0.039558583	0.10573756	0.013999773
18	0.032701795	0.047707206	0.109928841	0.013831839	0.033468147	0.0557696	0.112997157	0.014143088
19	0.03867147	0.041014082	0.101935546	0.016412085	0.034679956	0.046424977	0.098880706	0.017110783
20	0.038105431	0.048559571	0.108640287	0.015769151	0.049801057	0.056283104	0.117011461	0.016588262
21	0.063907425	0.063203897	0.139189976	0.015923771	0.049208265	0.05956841	0.116045094	0.015932046
22	0.052066221	0.044084623	0.104311937	0.013971005	0.051968437	0.043457376	0.102035851	0.014116573
23	0.035123503	0.040575156	0.119900833	0.01389219	0.035819264	0.039412581	0.112894416	0.013739912
24	0.042934953	0.092818744	0.129432637	0.020802683	0.041386799	0.103269988	0.13969608	0.020681479
25	0.054398411	0.062940182	0.119498227	0.016540453	0.043365502	0.064650574	0.114110023	0.014784476
26	0.048843926	0.053016444	0.108469117	0.017189545	0.042108071	0.048933722	0.10025114	0.015258728
27	0.047561549	0.041691355	0.105035351	0.016983474	0.059611861	0.042530857	0.107725948	0.019258246
28	0.042814987	0.058129858	0.106750374	0.016508899	0.046209304	0.06212401	0.114392163	0.017956363
29	0.03479514	0.039870226	0.09426786	0.014681532	0.03619633	0.038381127	0.093664896	0.015014396
30	0.04188021	0.05258487	0.10607014	0.017431581	0.040204205	0.0513622	0.103321629	0.018989933
31	0.061342614	0.063980166	0.139164855	0.025808722	0.064106573	0.064804901	0.142163929	0.027189509
32	0.057511198	0.075809897	0.134700509	0.034860312	0.058719716	0.079861812	0.14097025	0.03718838
33	0.05242671	0.074806079	0.139385942	0.021987878	0.115694723	0.084162807	0.146297849	0.046998908
34	0.053102737	0.041152962	0.10645457	0.014747579	0.053865957	0.042657917	0.111943448	0.014774801
35	0.036707299	0.059339649	0.112545956	0.012934064	0.034794878	0.068675161	0.123214321	0.013761018
36	0.034220821	0.060442787	0.124295576	0.014889853	0.050566799	0.128321204	0.223144086	0.024108752
37	0.040105075	0.038703409	0.093687241	0.01639284	0.041281079	0.045552142	0.09166976	0.016480091
38	0.032570333	0.070493674	0.13628127	0.013915279	0.031778926	0.076336573	0.145867671	0.013589548
39	0.037194313	0.121324164	0.166788224	0.016135311	0.034720844	0.11850375	0.161527616	0.016500818
40	0.040276968	0.055411009	0.113153528	0.017603304	0.047377522	0.054914194	0.13726871	0.021624189
41	0.065373936	0.058962788	0.127350867	0.018741878	0.078465243	0.078034483	0.142147402	0.023928861
42	0.064699614	0.041520436	0.112960574	0.014421077	0.038632672	0.043109221	0.097300434	0.013882141
43	0.058093362	0.084921664	0.169022188	0.020287841	0.073095227	0.106329949	0.201401949	0.024980028
44	0.03917478	0.049292608	0.111231454	0.013818342	0.040885408	0.048949419	0.102607617	0.01410096
45	0.03887148	0.04975969	0.116346461	0.014276553	0.041159877	0.045203802	0.120953633	0.014060799
46	0.039155778	0.047298132	0.10379784	0.014420736	0.034983971	0.061213643	0.107166964	0.014185942
47	0.049883807	0.048054069	0.125310859	0.016727015	0.049309491	0.045726689	0.123449483	0.017638312
48	0.050433909	0.054491905	0.125179975	0.022972591	0.107118341	0.076041062	0.164696266	0.040592594
49	0.054253588	0.079653986	0.097294065	0.015626663				
50	0.040711157	0.050707846	0.103700913	0.014231951	0.045904712	0.075040632	0.130277149	0.014765053
51	0.04291582	0.03635119	0.103773124	0.014112497	0.039680874	0.035324474	0.101701036	0.014033986
52	0.036686977	0.066867684	0.123738693	0.014011398	0.033217147	0.063159695	0.107045948	0.013617853
53	0.048615629	0.045098702	0.108452279	0.016014618	0.049410491	0.048131946	0.107855422	0.016198164
54		0.041639054	0.105524678	0.013943847		0.042848547	0.10786313	0.014429564
55	0.069914969	0.0672822	0.123460784	0.018659378	0.039123373	0.077958754	0.123669918	0.015750005
56	0.037021828	0.053882321	0.10811756	0.014495749	0.036258328	0.051104259	0.104014949	0.014457995
57	0.05287141	0.041761473	0.134655016	0.018145057	0.054699577	0.03966083	0.129438053	0.017422797
58	0.038586605	0.032945455	0.104262594	0.014395678	0.039683476	0.030166971	0.101640768	0.014628031
59	0.031813074	0.042424311	0.102297263	0.013412986	0.032040542	0.041563337	0.107994509	0.01409573
60	0.042851606	0.052636282	0.116363197	0.013223853				
61	0.0605336	0.0364841	0.09372175	0.014212844	0.0609912	0.029366309	0.082794155	0.014007104
62	0.046964809	0.041411202	0.102491154	0.020104396	0.050041703	0.04416679	0.10319228	0.024092461
63	0.047684987	0.041771368	0.103019898	0.015901115	0.057712185	0.047378596	0.102450569	0.017128752
64	0.036498084	0.038998498	0.11116833	0.016104744	0.034171464	0.037253725	0.109226356	0.014497921
65	0.039612536	0.05245603	0.105783653	0.01549345	0.030374571	0.049682088	0.10483862	0.013856499
66	0.032153945	0.054165312	0.109014027	0.014111302	0.028029784	0.049855583	0.102178417	0.013926154
67	0.065496011	0.030289974	0.086971835	0.018603436	0.070170507	0.02499985	0.092532325	0.019241835
68	0.041789899	0.048283838	0.101644825	0.014294027	0.041057223	0.046219467	0.103743442	0.014682984
69	0.04217966	0.037649939	0.107370462	0.014392263	0.042834811	0.038284896	0.110928961	0.014330875
70	0.028516559	0.051333608	0.101238503	0.014864313	0.028595058	0.050743425	0.104480838	0.014676391
71	0.032255844	0.04837045	0.10016516	0.014640666	0.038341195	0.049737229	0.102092685	0.015458465
72	0.032095843	0.040569688	0.104579268	0.014013525	0.032673437	0.037217405	0.102376089	0.014789801
73	0.044473455	0.045418237	0.094296369	0.014449501	0.038760593	0.034057279	0.090815326	0.014563129
74	0.036584647	0.040880306	0.104919869	0.01391925	0.033268192	0.02958096	0.104505303	0.014479627
75	0.043652808	0.063637862	0.132259176	0.013999681	0.04018518	0.066628977	0.143775216	0.012984994
76	0.058567315	0.076637996	0.100019971	0.019627271	0.058020696	0.070360509	0.090728715	0.019097881
77	0.043865429	0.053300881	0.111669923	0.018736773	0.050225992	0.065281452	0.117769029	0.023008193
78	0.055833769	0.074976936	0.152136434	0.020188418	0.061109116	0.17741878	0.326070566	0.01537373
79	0.062752495	0.034210263	0.106232817	0.017168701	0.065537416	0.038301983	0.104347804	0.020709859
80	0.050563315	0.037174016	0.101983248	0.015618256				
81	0.044006144	0.09166093	0.1448023	0.014317238	0.043363848	0.088717519	0.140859859	0.014350573
82	0.042054064	0.043743067	0.101693689	0.017555512	0.056434737	0.04716997	0.101361215	0.021224215
83	0.043598388	0.035168715	0.107201272	0.015351128	0.040186604	0.037659962	0.109097676	0.015670695
84	0.0358112	0.038681945	0.097847444	0.015439862	0.0354948	0.029215856	0.101366188	0.015087136
85	0.038171311	0.045671268	0.105190723	0.014956844	0.051618437	0.04948337	0.10819713	0.019382497
86	0.04416263	0.057105744	0.123153201	0.016720036	0.045321949	0.070498874	0.14649694	0.017411563
87	0.040756624	0.111313708	0.154879985	0.018552023	0.044418797	0.107656143	0.151951766	0.020169077
88	0.043430706	0.044573554	0.095152956	0.014973644	0.04035094	0.045314263	0.087712986	0.014708432
89	0.041106495	0.040481459	0.100438554	0.014104362	0.058342519	0.050524918	0.115756582	0.015570924
90	0.033242513	0.052287412	0.113105631	0.012850017	0.032327565	0.048601165	0.109492252	0.012694361
91	0.030204937	0.055423535	0.112610326	0.013021711				
92	0.041575166	0.050893478	0.109366631	0.014792186	0.037316097	0.064136314	0.128976273	0.014518811
93	0.046441358	0.04895401	0.114084301	0.016433317	0.058458066	0.056738709	0.128133358	0.017974332
94	0.050980841	0.041618379	0.101368396	0.015108426	0.532384861	0.093485918	0.126678178	0.070686319
95	0.045243707	0.038671666	0.100945682	0.018503416	0.051837176	0.050009747	0.123960249	0.023152523
96	0.032119461	0.046830841	0.098522762	0.014194562	0.03168476	0.045565907	0.097619924	0.014478599
97	0.061088404	0.037193169	0.103323087	0.014665324	0.044472585	0.047672761	0.128599815	0.014651996
98	0.034287994	0.05220052	0.11111127	0.016438501	0.031308646	0.047417781	0.108422078	0.012285524
99	0.042128057	0.044696206	0.114981383	0.017507181	0.04678882	0.049516937	0.118659082	0.018356302
100	0.037593034	0.039847872	0.107930664	0.013732757	0.036067114	0.039393984	0.108567653	0.013651467
101	0.034471572	0.029386376	0.107202797	0.014535835	0.033852968	0.030522612	0.101499054	0.01472642
102	-6.12245E+97	-6.38298E+97	0.10311362	-6.12245E+97			0.119185318	-1.81818E+98
103	0.039965398	0.046202924	0.110159315	0.014979087	0.035823232	0.049081977	0.109549772	0.017121608
104	0.044311408	0.088317181	0.156002791	0.017769649	0.041996819	0.085263414	0.150571816	0.018283828
105	0.029745034	0.038765297	0.09929386	0.019025283	0.031489684	0.038706037	0.101338391	0.02428949
106	0.042884871	0.028195603	0.097760693	0.015307523	0.044139501	0.026617375	0.098189708	0.015962252
107	0.032321474	0.042768559	0.105652406	0.013790893				
108	0.032989814	0.041652935	0.107570363	0.015260926	0.035336469	0.043423798	0.110196394	0.016250724
109	0.06243495	0.113068837	0.172242462	0.025040802	0.061984905	0.11443574	0.172079251	0.025229497
110	0.041950658	0.052397206	0.114425908	0.017655446	0.04368464	0.058095724	0.117541876	0.018113268
111	0.055999519	0.070253258	0.111601065	0.020431972	0.055999507	0.070803373	0.113841952	0.020639912
112	0.045571314	0.060591331	0.109669517	0.014879787	0.061979776	0.069186153	0.125293307	0.018250246
113	0.046458228	0.058465239	0.119755242	0.020261077	0.079361158	0.093354175	0.152881228	0.035875733
114	0.042285915	0.041095042	0.106314208	0.018618321	0.058541715	0.053611207	0.122696066	0.025906785
115	0.038022907	0.039424815	0.102678501	0.014783422	0.033966743	0.028528563	0.083813189	0.014739678
116	0.041494791	0.046094911	0.127557627	0.016532792	0.058746508	0.045741933	0.117658076	0.028937253
117	0.043307928	0.060836281	0.130480566	0.020086033	0.047897635	0.083404651	0.159073359	0.025091347
118	0.048527632	0.066669883	0.117590431	0.024786466	0.056772772	0.09128614	0.136336297	0.034742554
119	0.038715578	0.048526055	0.125943056	0.015314502	0.036505931	0.047655917	0.124021206	0.015070682
120	0.054411984	0.051009227	0.105065666	0.022597834	0.06203451	0.049901696	0.109269512	0.026736367
121	0.039299975	0.04133511	0.096557775	0.0161842	0.040456373	0.043077236	0.101553155	0.016448483
